# Generation and Amplification of Tunable Multicolored Femtosecond Laser Pulses by Using Cascaded Four-Wave Mixing in Transparent Bulk Media

**DOI:** 10.3390/s100504296

**Published:** 2010-04-29

**Authors:** Jun Liu, Takayoshi Kobayashi

**Affiliations:** 1 Department of Applied Physics and Chemistry and Institute for Laser Science, University of Electro-Communications, Chofugaoka 1-5-1, Chofu, Tokyo 182-8585, Japan; E-Mail: jliu@ils.uec.ac.jp; 2 International Cooperative Research Project (ICORP), Japan Science and Technology Agency, 4-1-8 Honcho, Kawaguchi, Saitama 332-0012, Japan; 3 Department of Electrophysics, National Chiao Tung University, 1001 Ta Hsueh Rd. Hsinchu 300, Taiwan; 4 Institute of Laser Engineering, Osaka University, Yamadakami 2-6, Suita, Osaka 565-0871, Japan

**Keywords:** femtosecond spectroscopy, CFWM, XPM, FOPA, tunable laser pulse, pulse compression

## Abstract

We have reviewed the generation and amplification of wavelength-tunable multicolored femtosecond laser pulses using cascaded four-wave mixing (CFWM) in transparent bulk media, mainly concentrating on our recent work. Theoretical analysis and calculations based on the phase-matching condition could explain well the process semi-quantitatively. The experimental studies showed: (1) as many as fifteen spectral up-shifted and two spectral down-shifted sidebands were obtained simultaneously with spectral bandwidth broader than 1.8 octaves from near ultraviolet (360 nm) to near infrared (1.2 μm); (2) the obtained sidebands were spatially separated well and had extremely high beam quality with M^2^ factor better than 1.1; (3) the wavelengths of the generated multicolor sidebands could be conveniently tuned by changing the crossing angle or simply replacing with different media; (4) as short as 15-fs negatively chirped or nearly transform limited 20-fs multicolored femtosecond pulses were obtained when one of the two input beams was negatively chirped and the other was positively chirped; (5) the pulse energy of the sideband can reach a μJ level with power stability better than 1% RMS; (6) broadband two-dimensional (2-D) multicolored arrays with more than ten periodic columns and more than ten rows were generated in a sapphire plate; (7) the obtained sidebands could be simultaneously spectra broadened and power amplified in another bulk medium by using cross-phase modulation (XPM) in conjunction with four-wave optical parametric amplification (FOPA). The characterization showed that this is interesting and the CFWM sidebands generated by this novel method have good enough qualities in terms of power stability, beam quality, and temporal features suited to various experiments such as ultrafast multicolor time-resolved spectroscopy and multicolor-excitation nonlinear microscopy.

## 1. Introduction

Ultrafast time-resolved spectroscopy is a powerful technique for the investigation of electronic and vibrational dynamics in molecules, which are key elements in various fields in physics, chemistry, biology, and materials science research [1–7], mainly because this research can provide important information of electronic relaxation and dynamics in molecular vibrations [1–7]. The primary process of these dynamics ocurrs in the femtosecond to picosecond time regime. To investigate these processes by time-resolving the dynamics, the laser pulse in the experiment must have shorter duration than the time range of the phenomena of interest. Wavelength-tunable laser pulses with durations shorter than 20-fs are needed to investigate the electronic and vibrational dynamics of molecules in a wide range of applications in the fields of photochemistry, photophysics, and photobiology [1–7]. There are even wider application of the ultrashort pulses are proposed and used in non-photo chemistry, physics, and biology. They are for example impulsive excitation of coherent vibration in the ground state dynamics, plasma diagnosis, and multiphoton microscopy. In some applications, several multicolored ultrashort pulses are needed in some spectroscopy experiments, for example multicolor pump-probe experiments [8], femtosecond CARS spectroscopy [9] and two-dimensional spectroscopy [10]. In other applications than spectroscopy such as nonlinear microscopy, tunable multicolored femtosecond pulses excitation also shows lots of advantages [11–13].

There have been ongoing efforts to develop new and convenient methods for producing tunable ultrashort laser pulses. Over the past decade, tunable femtosecond laser systems with microjoule pulse energies from UV to mid-IR spectral region have been developed based on three-wave mixing in nonlinear crystals [14–18]. Using noncollinear optical parametric amplifier (NOPA), tunable few-cycle pulses were generated and had been widely used in pump-probe experiments [3–7]. On the other hand, four-wave mixing (FWM) has been studied in many various media and extensively used in a wide range of fields [19]. Recently, FWM has been studied in various optically transparent media as a new generation method of tunable ultrashort pulses covering an ultrabroadband range [20–53]. Tunable visible ultrashort pulses were generated by FWM through filamentation in a gas cell [20]. Femtosecond pulses in deep UV and mid-IR have also been generated by FWM through filamentation [21,22]. Several different wavelengths also in UV were generated owe to CFWM in a hollow fiber filled with noble gases [23,24].

It was discovered that ultrabroadband spectra and tunable ultrashort pulses could also be generated in various transparent bulk media using FWM process [25–52]. In the case of solid-state bulk media, due to high material dispersion, phase matching can be obtained only if the two pump beams have a finite crossing angle in the media. Tunable mid-IR pulses could be obtained in the range 2.4 to 12 μm using FWM in CaF_2_ and BaF_2_ plates [25,26]. Separated CFWM multicolored sidebands were generated in a BK7 glass [27–29], a fused silica glass [30–33], and a sapphire plate [34,35] with two intersecting femtosecond laser beams. Sidebands with spectrum extending over more than 1.5 octaves from the UV to the near-IR were obtained. Different-frequency resonant FWM, known as cascaded stimulated Raman scattering (SRS) or coherent anti-Stokes Raman scattering (CARS), have been demonstrated, and sidebands with broadband spectra have been generated in many nonlinear crystals including PbWO_4_ [36,37], LiNbO_3_ [38], KNbO_3_ [39], TiO_2_ [40], KTaO_3_ [41,42], YFeO_3_ [43], SrTiO_3_ [44], diamond [45], and BBO [46]. As many as 20 anti-Stokes (AS) and two Stokes (S) coherent sidebands were generated with high conversion efficiency by focusing dichromatic ultrashort pulses into a Raman-active crystal, lead tungstate (PbWO_4_) [36,37]. A pair of isolated 25 fs and 13 fs pulses was obtained in a LiNbO_3_ crystal and KTaO_3_, respectively, by combining these sidebands into a single beam at room temperature [38,42]. On the other hand, highly efficient high-energy noncollinear FOPA was achieved in a transparent bulk Kerr medium by using another intense pulse pump [47–50]. Optical amplifications using FOPA were also demonstrated in UV and near-infrared spectral range [51–53].

In the article, we review our recent research progress on the generation and amplification of wavelength-tunable multicolored femtosecond laser pulses by using CFWM in transparent bulk media [30–35,46,50]. The article is organized as follows: in Section 2 we first explain the CFWM process with a phase-matching configuration. In Section 3, the experimental setup of our laser system is firstly presented in Section 3.1. We then discuss the generation of wavelength-tunable multicolored femtosecond laser pulses in a fused silica plate by changing the crossing angle or by replacing the transparent bulk media in Section 3.2. In Section 3.3, the pulse energy of the generated sidebands is upgraded to a μJ level. In Section 3.4, we describe the generation of 15-fs negatively chirped or nearly transform-limited 20-fs multicolor femtosecond pulses by using chirped incident pulses. Then we show the characterization of beam qualities and power stabilities of generated sidebands in Section 3.5. The CFWM process in a BBO crystal and a sapphire plate are also demonstrated in Section 3.6, where the sidebands can be generated on both sides of the input beams. In Section 3.7, we describe the phenomenon that broadband 2-D multicolored arrays with more than ten periodic columns and more than ten rows are generated in a sapphire plate. Finally in Section 3.8, we show the simultaneous compression and amplification of the generated sidebands in another bulk medium by using XPM in conjunction with FOPA. In Section 4, some theoretical calculation based on the phase-matching condition is done to explain the process semi-quantitatively. Finally, we conclude the article and give some prospects for future research directions in Section 5.

## 2. Theoretical Analysis of CFWM Process

**Figure 1.**
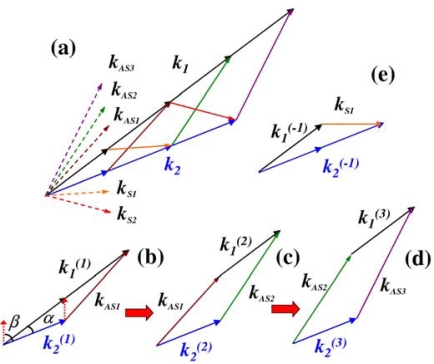
(a) Phase-matching geometry for CFWM process. Phase-matching geometries for generating (b) AS1, (c) AS2, (d) AS3, and (e) S1. ***k***_1_ and ***k***_2_ are the two input beams. The angle α is the crossing angle between the two input beams in the medium [33].

CFWM processes based on the phase-matching condition are shown schematically in Figure 1(a). The wave vectors ***k*_1_** and ***k*_2_** are corresponding to the two input beams with respective frequencies of *ω1*, *ω2* (*ω1* > *ω2*). The CFWM can be disassembled step by step as follows: to simplify the expressions, we named the *m*-order spectral up-shifted and down-shifted sidebands as ASm and Sm, respectively. In the first step, two ***k^(1)^*_1_** photons and one ***k^(1)^*_2_** photon generate a first-order spectral up-shifted (named first-order anti-Stokes(AS1)) photon ***k*_AS1_**; the phase-matching condition of this FWM process can be described as ***k*_AS1_** = **2*k^(1)^*_1_** − ***k^(1)^*_2_** and *ω*_*AS*1_ *=* 2*ω_1_*^(1)^ − *ω*_2_^(1)^, as shown in Figure 1(b). Here, ***k^(1)^*_1_** and ***k^(1)^*_2_** are the wave vectors of the photons with the corresponding wavelength satisfying the phase matching condition and frequency relation in the first FWM process. Then, succeeding FWM process among the generated ***k*_AS1_** photon, one ***k^(2)^*_1_** photon and one ***k^(2)^*_2_** photon generate a second-order anti-Stokes photon ***k*_AS2_**. It is needed to note that the frequencies and wave vectors of ***k^(2)^*_1_** photon and ***k^(2)^*_2_** photon in this step are different from ***k^(1)^*_1_** photon and ***k^(1)^*_2_** photon that generated ***k*_AS1_** photon. The phase-matching condition of this second step of CFWM process is given by ***k*_AS2_** = ***k*_AS1_** + ***k^(2)^*_1_** − ***k^(2)^*_2_** ≈ 3***k^(1)^*_1_** − 2***k^(1)^*_2_**, in which the directions of ***k^(2)^*_1_** and ***k^(2)^*_2_** are the same as those in ***k^(1)^*_1_** and ***k^(1)^*_2_** but their modulus values and hence the wavelengths are different. Because of the differences in wavelengths of ***k^(2)^*_1_** and ***k^(2)^*_2_** in the second process, the value of ***k*_AS2_** is slightly different from 3***k^(1)^*_1_** − 2***k^(1)^*_2_**. Therefore the value of ***k*_AS2_** is given only approximately by ***k*_AS2_** ≈ 3***k^(1)^*_1_** − 2***k^(1)^*_2_** and the frequency is given by *ω*_*AS*2_ = *ω*_*AS*1_ + *ω*_1_^(2)^ − *ω*_2_^(2)^ ≈ 3*ω*_1_^(1)^ − 2*ω*_2_^(1)^, as shown in Figure 1(c). Subsequently, as shown in Figure 1(d), one ***k*_AS2_** photon together with one ***k^(3)^*_1_** photon and one ***k^(3)^*_2_** photon generate a ***k*_AS3_** photon; the phase-matching condition is given by ***k*_AS3_** = ***k*_AS2_** + ***k^(3)^*_1_** − ***k^(3)^*_2_** ≈ 4***k^(1)^*_1_** − 3***k^(1)^*_2_**, *ω_AS_*_3_ = *ω*_*AS*2_ + *ω*_1_^(3)^ − *ω*_2_^(3)^ ≈ 4*ω*_1_^(1)^ − 3*ω*_2_^(1)^. To simplify the expression, the *m*th-order anti-Stokes sideband has the following phase-matching condition: ***k*_AS*m*_** = ***k*_AS(*m−1*)_** + ***k^(m)^*_1_** − ***k^(m)^*_2_** ≈ (*m* + 1)***k^(1)^*_1_** − *m****k^(1)^*_2_**, *ω*_*AS*m_ ≈ (m+1)*ω*_1_^(1)^ − m*ω*_2_^(1)^. We need keep in mind that in every step, even the direction of ***k^(m)^*_1_** photon and ***k^(m)^*_2_** photon are the same, the values of frequency and wave vector length *ω*_1_^(m)^, *ω*_2_^(m)^, |***k^(m)^*_1_**|, and |***k^(m)^_2_***| are different in every FWM step of *m*. On the spectral down-shifted side, two ***k^(−1)^*_2_** photons with one ***k^(−1)^*_1_** photon generate a first-order Stokes photon ***k*_S1_** (named S1), as shown in Figure 1(e). Then the generated ***k*_S1_** photon together with a ***k^(−2)^*_1_** photon and a ***k^(−2)^*_2_** photon generate a second-order Stokes photon ***k*_S2_**. Therefore, the *m*th-order Stokes sideband will have the following phase-matching condition: ***k*_S*m*_** = ***k*_S(*m−1*)_** + ***k^(−m)^*_2_** − ***k^(−m)^*_1_** ≈ (*m* + 1)***k^(−1)^*_2_** − *m****k^(−1)^*_1_**, *ω_Sm_* ≈ (m + 1)*ω*_2_^(−1)^ − m*ω*_1_^(−1)^. Thus, all the processes are FWM processes with third-order nonlinearity. Higher-order signals are obtained from the generated adjacent lower-order signals; hence the process is called *cascaded* FWM.

Figure 1 shows that the required length of wave vector |***k^(m)^***| for the generation of a FWM signal increases on both the anti-Stokes and Stokes sides when the order number increases. For anti-Stokes beams, the wavelength is blue-shifted as the order number increases, which accords with the need of increasing length of |***k^(m)^***|. In this case, phase matching can be achieved up to higher-order sidebands. However, the wavelengths of the Stokes beams increase with an increase of the order number. This red-shifted wavelength does not satisfy the requirement of increasing length of |***k^(−m)^***|. As a result, the phase mismatch for the Stokes signal increases rapidly with the order number. Consequently, there are fewer Stokes sidebands than anti-Stokes sidebands generated in the output signal. The calculation described below gives the same result. We also can see that the maximum exit angle in the medium will be limited by the angle *β* (see Figure 1(b)) on the anti-Stokes side due to the phase-matching condition. Based on the trigonometric formula, *β* can be expressed as: $\beta =\alpha +{\text{sin}}^{-1}\;(\left|k_{2}\right|\text{sin}\;\alpha /\sqrt{{\left|k_{1}\right|}^{2}+{\left|k_{2}\right|}^{2}-2\left|k_{1}\right|\left|k_{2}\right|\text{cos}\;\alpha )}$, where *α* is the crossing angle between the two incident beams in the medium (see Figure 1(b)).

As for the generated signals, there are many routes for generating every sideband including the backward process as seen from Figure 1(a). In principle, every closed loop that includes the generated signal vector is a route. Take ***k*_AS2_** as an example; there are many possible routes for generating ***k*_AS2_**: ***k*_AS2_** = ***k*_AS1_** + ***k^(2)^*_1_** − ***k^(2)^*_2_**, ***k*_AS2_** = ***k^(1)^*_1_** + ***k^(2)^*_1_** − ***k*_S1_**, ***k*_AS2_** = ***k^(1)^*_1_** + ***k^(1)^*_1_** + ***k^(2)^*_1_** − ***k^(1)^*_2_** − ***k^(2)^*_2_**, ***k*_AS2_** = ***k^(3)^*_2_** + ***k*_AS3_** − ***k^(3)^*_1_**, and so on. In a medium, intrinsic fifth- and higher-order nonlinear processes are frequently ignored since the effects are much smaller than that third-order nonlinearity process. As described above, the higher-order Stokes signal is very weak due to the large phase mismatch and also because the crossing angle between the higher-order Stokes beam and the anti-Stokes beam is very large, the interaction length is short. Therefore, processes due to higher-order Stokes signals can also be ignored. Thus, the *m*th-order anti-Stokes signal is mainly generated by the cascading ***k*_AS*m*_** = ***k*_AS(*m−1*)_** + ***k^(m)^*_1_** − ***k^(m)^*_2_** process. In the process, the contribution from the feedback process ***k*_AS*m*_** = ***k*_AS(*m + 1*)_** − ***k^(m)^*_1_** + ***k^(m)^*_2_** is small and can be neglected for higher order signals. However, it is necessary to consider the contribution from the strong first-order Stokes or anti-Stokes signals for low-order anti-Stokes or Stokes signals.

## 3. Experimental Section

### 3.1. Experimental Setup

**Figure 2.**
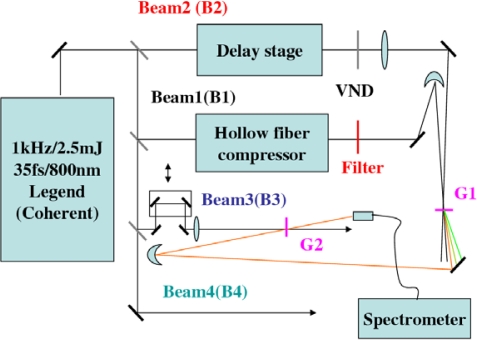
Experimental setup. VND: variable neutral-density filter. Filter: BPF at 700 nm center wavelength with 40 nm bandwidth, or SPF with cut-off wavelength at 800 nm or 820 nm, or long wavelength pass filter (LPF) with cut-off wavelength at 850 nm. G1:1mm-thick fused silica plate or other nonlinear media. G2: 0.5-mm-thick fused silica plate.B1, B2, B3, B4: please refer to the main text in the paper [50].

The experiments reported throughout this article were performed using a 1-kHz Ti:sapphire regenerative amplifier laser system (Micra + Legend − USP, Coherent). The system generates 35 fs pulses of about 2.5 mJ per pulse at a repetition rate of 1-kHz with the central wavelength at 800 nm. Figure 2 showed the experimental setup. The laser pulse was split into four beams. A 300 μJ beam (called beam1) was focused into a hollow-core fused silica fiber with 250-μm inner diameter, 3-mm outer diameter, and 60-cm length that filled with krypton gas. The pulse after hollow-core fiber has a broadband spectrum extending from 600 to 950 nm and has the pulse energy of about 190 μJ. The broadband spectrum was dispersion compensated using a chirped mirror pair and a pair of glass wedges. By changing the bounce times on the chirped mirror and tuning insert thickness of the glass wedge, we can obtained nearly transform limited pulse, negatively chirped pulse, and positively chirped pulse for following different experiments. The obtained shortest pulse duration after hollow fiber compression system was about 10 fs. After passing through a spectral filter (BPF1: band-pass filter (BPF) at 700 nm center wavelength with 40 nm bandwidth, or short wavelength pass filter (SPF) with cut-off wavelength at 800 or 820 nm, or long wavelength pass filter (LPF) with cut-off wavelength at 850 nm), beam1 was focused into a transparent bulk medium (G1:1-mm-thick fused silica glass plate or other nonlinear media) by a concave mirror with a focal length of 600 cm. Another beam (called beam2) with pulse energy about 100 μJ passed through a delay stage having less than 3 fs resolution. Beam2 was first attenuated by a variable neutral density (VND) filter and it was then focused into the transparent bulk medium by a lens with a focal length of 1 m. In the XPM in conjunction with FOPA experiment, the third beam (called beam3) with about 160 μJ was used as the pump pulse. The beam was focused by a 1-m focal length lens onto a 0.5-mm-thick fused silica plate (G2) after passing through a VND filter and a motor-driven delay stage with a resolution of 10-nm/step. The fourth beam (called beam4) was used to measure the pulse duration by the cross-correlation frequency resolved optical gating (XFROG) technique [54] by mixing it with the two incident beams or the generated sidebands in a 10-μm-thick BBO crystal.

In the experiment, the diameters of the incident beams on the glass plate and the generated sidebands were measured using a CCD camera (BeamStar FX33, Ophir Optronics). The pulse durations of the two input beams before the transparent bulk medium and the generated sidebands were measured using XFROG technique. The spectral and temporal intensity profiles and the spectral and temporal phase were retrieved using the commercial software, FROG 3.0 from Femtosoft Technologies. An optical fiber was used to pick up the incident beams or different order sidebands to measure their spectra using a multichannel spectrometer (USB4000, Ocean Optics). The polarizations of the input beams were checked to be parallel polarization in the experiment if there was no description. The beam intensities on the glass surface were nearly one order of magnitude lower than the optical breakdown threshold for all of the transparent bulk media used and no damage was found during or after irradiation by the beams in all experiments. The position of beam2 was fixed and the crossing angle between beams1 and beam2 was varied with a precision of ±0.02° by adjusting the position of beam1 on the surface of the concave mirror in front of the transparent bulk medium.

### 3.2. Generation of Wavelength-Tunable Multicolored Sidebands

#### 3.2.1. Generation of wavelength-tunable sidebands by changing crossing angle

**Figure 3.**
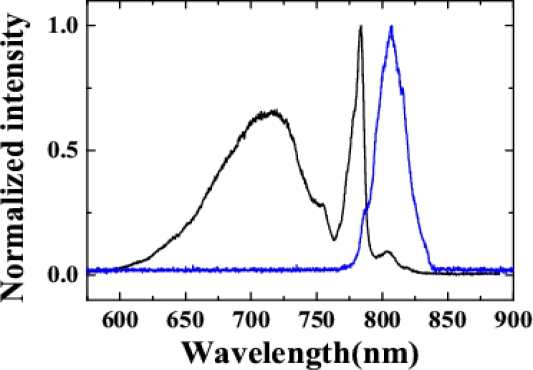
The spectra of the input beams prior to the fused silica glass plate. The black curve plots spectrum of beam1, while the blue curve plots spectrum of beam2.

In the experiment, a 1-mm-thick fused silica plate was used as the nonlinear medium (G1) to generate CFWM sidebands. The cross-correlation width of beam2 with beam4 was about 82 fs. The cross-correlation width of beam1 with beam4 was extended to about 165 fs with positive chirp. The diameters of the two input beams on the surface of the fused silica glass were measured to be 180 μm. A SPF with cut-off wavelength at 800 nm was used as the filter for beam1. The spectra of beam2 and beam1 in front of the fused silica glass plate are shown in Figure 3.

**Figure 4.**
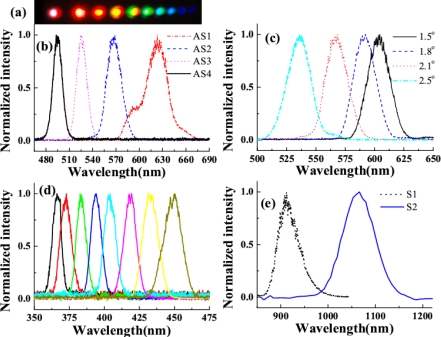
(a) Photograph of sidebands on a sheet of white paper 1 m after the glass plate. (b) The spectra of the sidebands from AS1 to AS4 when the crossing angle was 2.1°. (c) The spectra of AS2 at four different crossing angles of 1.5°, 1.8°, 2.1°, and 2.5°. (d) Spectra of high-order anti-Stokes from AS15 to AS8 and (e) two Stokes signals when the crossing angle was about 1.5° [30].

As beam1 and beam2 were focused on the fused silica glass synchronously in time and well overlapped in space, CFWM signals at different wavelengths were generated separately in space beside the input beams. Figure 4(a) shows a photograph of the CFWM sidebands on a white sheet of paper placed about 1 m after the glass plate. The first two beams on the left side were the two input beams, beam2 and beam1 from the left to the right, respectively. In the experiment, the input powers of beam1 and beam2 were 11 and 19 mW, respectively. Figure 4(b) shows the spectra of CFWM sidebands from AS1 to AS4 when the crossing angle between the beam1 and beam2 was 2.1°. It can be seen that the spectra extend from 475 to 675 nm. Each spectrum has a nice Gaussian profile and about 600 cm^−1^ bandwidth, which supports a transform-limited pulse duration of about 25 fs. It can be seen from Figure 4(c) that the peak wavelength of AS2 can be tuned from 500 to 625 nm by changing the crossing angle between the two input beams from 1.5° to 2.5°. When we refer to Figure 4(b), it is obvious that this tuning range fully covers the wavelength range of AS1 and AS3. Thus, the spectra of the sidebands are able to be continuously tuned in a broadband range by changing the crossing angle.

In the experiment, as many as fifteen anti-Stokes signals and two Stokes signals with the wavelength extending from 360 nm to 1.2 μm, corresponding to more than 1.8 octaves, were obtained. For clarity, only the spectra of the high-order anti-Stokes and the two Stokes signals (S1 and S2) are shown in Figure 4(d) and Figure 4(e). This means that the sidebands can be tuned from UV to NIR in the broadband wavelength. Even though the pulse energy of the high order anti-Stokes was low, the broadband spectrum is still of great interest. These broadband sidebands can be used to obtain near single cycle pulse chain through Fourier synthesis of the sidebands [28].

**Figure 5.**
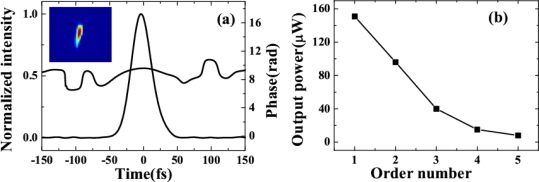
(a) The XFROG retrieved trace of the temporal profile and phase of AS1. The pulse duration is about 35 fs. The inset is the measured XFROG pattern; (b) the output power of AS1 to AS5 when the input power of beam1 and beam2 were 11 and 19 mW, respectively, and the crossing angle was 1.8° [30].

The pulse durations of AS1 and AS2 were measured by cross-correlation with beam4. We decrease the pulse duration of beam1 by changing the dispersion compensation. We found that the pulse duration of AS1 was even shorter than that of the two incident pulses, when the cross-correlation width of beam1 and beam4 was about 84 fs. Figure 5(b) shows the retrieved XFROG pulse trace and phase of the AS1 with a retrieved error of 0.017. The retrieved pulse duration was about 35 fs. The retrieved phase shows that there is some positive chirp in the pulse due to the positive chirped input pulses and the dispersion of the glass.

The output power of the sidebands was monitored by a silicon sensor power meter. The output power decreased from 153 to 8 μW as the order of the sidebands increased from AS1 to AS5 when the input power of beam1 and beam2 were 11 and 19 mW, respectively, and the crossing angle was 1.8°, as shown in Figure 5(b).

#### 3.2.2. Generation of wavelength-tunable sidebands by simply replacing the bulk medium

From the theory analysis in the second section, the wavelength of the generated sideband is decided by the phase-matching condition. As a result, it can also be tuned by changing the refractive index of the bulk medium. In the experiment, we change the refractive index of the medium by simply replacing the medium with different materials. The spectral filter used in the optical layout was a BFP centered at 700 nm with 40 nm bandwidth. The pulse duration of beam2 was 75 fs with positive chirp, while that of beam1 was 50 fs with negative chirp. The input power of beam1 (beam2) was 8 (24) mW and its diameter on the surface of the transparent bulk medium was 250 μm (300 μm) in the vertical direction and 300 μm (550 μm) in the horizontal direction.

**Figure 6.**
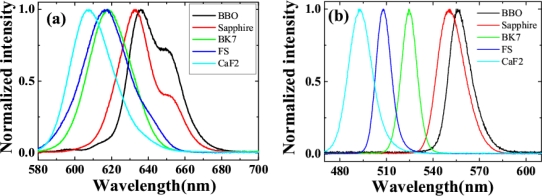
Spectra of (a) the AS1 sideband (c) the AS3 sideband of CaF_2_, fused silica, BK7, sapphire plate, and BBO crystal at 1.8° crossing angle [33].

We replaced the fused silica plate with other transparent bulk media: a CaF_2_ plate, a BK7 glass plate, a sapphire plate, and a BBO crystal cut at θ = 20.4°, ϕ = 0° (o light) all with a thickness of 1 mm. When the two input beams were identical and the crossing angle was fixed at 1.8°, the spectra for the same order sideband differed greatly for different transparent bulk media. Figure 6(a) shows the spectra of the AS1 signals for transparent bulk medium of CaF_2_, fused silica, BK7, sapphire plate, and BBO crystal. The signal generated by CaF_2_ has the shortest wavelength among them, while the BBO crystal induces the longest wavelength signal. Figure 6(b) shows the spectra of the AS3 signals of CaF_2_, fused silica, BK7, sapphire plate, and BBO crystal. The wavelength ranges of different-order signals from different bulk media overlap with each other. This means it is possible to continuously tune the spectrum by simply replacing the medium.

Table 1 shows the output power of different sidebands for several different bulk media when the external crossing angle is about 1.8°. In the case, the input powers of beam1 and beam2 were 6.5 and 25 mW, respectively. Even the output power of AS1 was the lowest for CaF_2_, the powers of AS2 and AS3 were still very high. For BBO, the power of the sideband decreased quickly as the order number increased. This output power dependence on the materials was almost the same as that of dependence on the external crossing angle as shown in Figure 9(b) to be discussed in the following Section 3.3. In the case, the angle of 1.8° is a large crossing angle in the case of CaF_2_ and a small angle in the case of BBO with respect to that in Figure 9(b).

### 3.3. Improvement of the Sideband Pulse Energy up to a uJ Level

**Figure 7.**
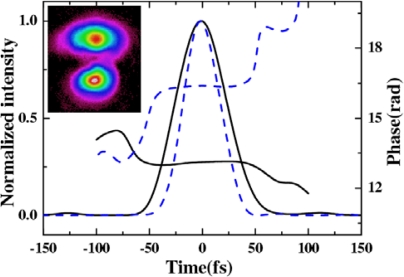
The temporal profile and phase of beam1 (dashed lines) and of beam2 (solid lines). The inset patterns are the spatial pattern beam1 (lower) and beam2 (upper) on the surface of the fused silica glass [31].

In the above experiment, the output power of the sideband was usually less than 1 μJ. To improve output pulse energy of the sideband, we optimized the overlapping of the spatial, temporal, and spectral parameters of the two input beams. In this experiment, the filter used in the optical layout was the BPF filter centered at 700 nm. A 1-mm thick fused silica plate was used as the nonlinear medium. The pulse durations of beam1 and beam2 were measured by XFROG with beam3 to be 40 ± 3 and 55 ± 3 fs, respectively, as shown in Figure 7. The nearly equal pulse durations of the two input pulses demonstrates that they were temporally well overlapped. The spatial mode of the two input beams on the surface of the fused silica glass was measured using a CCD camera, as shown in the inset of Figure 7. The beam widths of beam2 were 500 and 300 μm in the horizontal and vertical directions, respectively. For beam1, the beam widths were 440 and 285 μm in the horizontal and vertical directions, respectively. These elliptical cross sections were obtained by adjusting the beams to the edges of the lens and concave mirror. Owing to their elliptic shapes, the two input beams can be made to overlap strongly in the medium even if there was a crossing angle between them.

**Figure 8.**
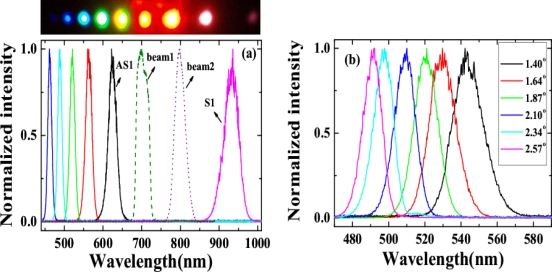
**(a)** The spectra of the sidebands from S1 through AS5 and of the two input beams when the crossing angle between the two input beams was 1.87°. **(b)** Spectra of AS3 at crossing angles of 1.40°, 1.64°, 1.87°, 2.10°, 2.34°, and 2.57°. The photograph at the top of Figure 8(a) shows the sidebands on a sheet of white paper placed 30 cm after the glass plate when the crossing angle between the two input beams was 1.87°. The first, second, and third spots from the right edge are S1, beam2, and beam1, respectively [31].

Multicolored CFWM signals appeared individually in space beside the two input beams as beam1 and beam2 were synchronously on the fused silica glass in both time and space. The photograph at the top of Figure 8(a) shows CFWM sideband signals on a white sheet of paper placed about 30 cm after the glass plate. The input powers of beam1 and beam2 were 9 and 20 mW, respectively. Figure 8(a) shows the spectra of the sideband wavelengths from S1 to AS5 CFWM signals along with the spectra of two input beams when the crossing angle between them was 1.87°. The spectrum extends from 450 to 1,000 nm. All the sidebands have nice Gaussian spectral profiles. According to Figure 8(b), the peak wavelength of AS3 can be tuned from 490 to 545 nm by changing the crossing angles from 1.40° to 2.57°. Compared with results in Section 3.2.1, this tuning range is narrower, due to the narrower spectral bandwidth of beam2.

**Figure 9.**
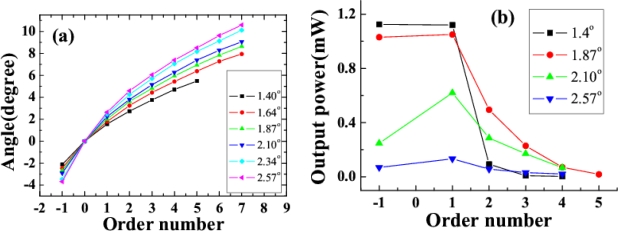
(a) Dependence of the angles between the generated sidebands and beam2 ( with 0 order number) on the order number of the sidebands when the crossing angle between the two input beams was 1.40°, 1.64°, 1.87°, 2.10°, 2.34°, and 2.57°. (b) Dependence of the output power on the order number when the crossing angle between the two input beams was 1.40°, 1.87°, 2.10°, or 2.57°. Order number-1 refers to S1, 1 refers to AS1, and so on [31].

A white screen was located 30 cm after the fused silica glass in order to mark the position of each sideband for different crossing angles. In this way, the crossing angles between the generated sidebands and beam2 were recorded, as plotted in Figure 9(a). The crossing angles between adjacent sidebands decreased as the order number increased for a given crossing angle between the input beams. Furthermore, the angle between each of the sidebands and beam2 increased with increasing of the crossing angle between the two input beams.

The output power of AS1 and S1 were 1.03 and 1.05 μJ, respectively, when the crossing angle between the two input beams was 1.87° and the input powers of beam1 and beam2 were 9 and 20 mW, respectively. The output power of sidebands depends on the crossing angle between the two input beams and the order number of the sidebands. Figure 9(b) shows the dependence of the output power on order number when the crossing angle between the two input beams was 1.40°, 1.87°, 2.10°, and 2.57°. The output power of S1 and AS1 decreased rapidly as the crossing angle between the two input beams increased from 1.40° to 2.57°. For a smaller crossing angle between the two input beams, say 1.40°, the output power of the sidebands decreased quickly with increasing order number. However, for a larger crossing angle between the two input beams, for example 2.57°, the output power of the sidebands slowly decreased as the order number increased. The energy conversion efficiency from the two input beams to sidebands was about 10% when the crossing angle was 1.87°.

**Figure 10.**
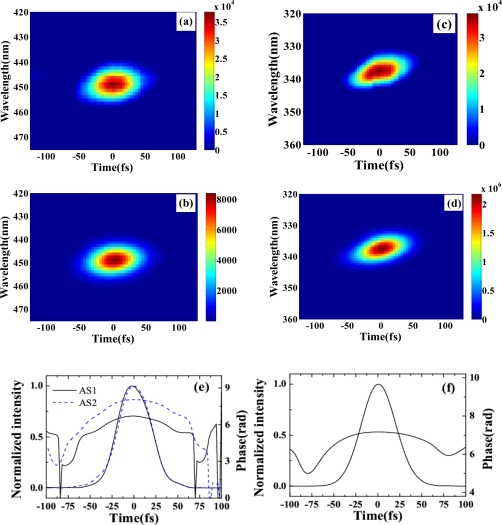
(a) Measured and (b) retrieved XFROG traces of S1; (c) measured and (d) retrieved XFROG traces of AS2 when the crossing angle was 1.87°. (e) Recovered intensity profiles and phase of AS1 (solid line) and AS2 (dashed line) (f) Recovered pulse profile and phase of S1 [31].

The pulse durations of S1, AS1, and AS2 were determined by cross-correlation measurement with beam4. Figures 10(a) and 10(b) show the measured and retrieved XFROG traces of S1 when the crossing angle was 1.87°. The corresponding XFROG traces of AS2 are shown in Figure 10(c) and 10(d). The recovered intensity profiles and phases of AS1 and AS2 are depicted in Figure 10(e) with retrieval errors of 0.012 and 0.010, respectively. The pulse durations of AS1 and AS2 were thereby found to be 45 ± 3 and 44 ± 3 fs, respectively. Figure 10(f) shows the recovered pulse profile and phase of S1 with a retrieval error of 0.005. The pulse duration was 46 ± 3 fs. All of the generated sidebands have almost similar pulse duration. The retrieved phase shows that there was some chirp in the pulses due to the small positive chirp of the input pulses and the dispersion of the glass.

### 3.4. Generation of Sub-20fs Sidebands Using Chirped Incident Pulses

In the above experiments, the output pulse duration was usually more than 30 fs with some positive chirp. To compensate this positive chirp and obtain much shorter pulse duration, it needs complex compensation system, which will also induce energy loss. In this section, negatively chirped or nearly transform-limited output pulses could be obtained when one of the pump beams was negatively chirped and the other was positively chirped. A 1-mm-thick fused silica plate was used as the nonlinear medium in this experiment.

#### 3.4.1. Principle of the experiment

**Figure 11.**
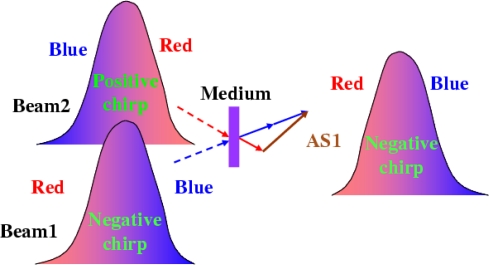
Schematic mechanism of negative CFWM sideband generation with positively and negatively chirped input pulses [32].

The principle behind the idea is simply explained in Figure 11. Beam1 is the input beam of shorter wavelength and is negatively chirped. Beam2 is another input beam having a longer wavelength and is positively chirped. As a result, when the two input beams are focused into a nonlinear medium to generate CFWM sidebands, the leading edges of the up-shifted anti-Stokes sidebands (AS1) will have shorter wavelengths, while the down-shifted Stokes sidebands (S1) will have longer wavelengths. Therefore, the anti-Stokes (Stokes) sidebands will be negatively (positively) chirped and the spectral bandwidth of the anti-Stokes sidebands will thereby be broadened.

The principle of the process can be easy understood using following expressions. In the CFWM process, the *m*th-order anti-Stokes sideband should has a phase matching condition: ***k***_ASm_ = ***k***_AS(*m*−1)_ + ***k***_1_ − ***k***_2_ = (*m* + 1)***k***_1_ − *m****k***_2_, *ω_ASm_* ≈ (*m* + 1)*ω*_1_ − *mω*_2_. Both chirped input pulses can be written as *E_j_*(*t*) ∝ exp {*i*[*ω*_*j*0_*t* + ϕ*_j_*(*t*)]}, *j* = 1,2, where beam1 was negatively chirped (∂^2^ϕ_1_(*t*)/∂*t*^2^ < 0) and beam2 (∂^2^ϕ_2_(*t*)/∂*t*^2^ > 0) was positively chirped.

The *m*-th (*m* > 0) order anti-stokes signal can be expressed as:

$$E_{\text{AS}m}\;(t)\propto \;\text{exp}\{i[((m+1)\omega_{10}-{m\omega }_{20})t+((m+1)\phi_{1}(t)-{m\phi }_{2}(t))]\}$$

Given (∂^2^ϕ_1_(*t*)/∂*t*^2^ < 0) and (∂^2^ϕ_2_(*t*)/∂*t*^2^ > 0), we can obtain

$$\partial^{2}\phi_{\mathrm{ASm}}\;(t)/\partial t^{2}=(m+1)\partial^{2}\;\phi_{1}\;(t)/{\partial t}^{2}-{m\partial }^{2}\phi_{2}\;(t)/{\partial t}^{2}<0$$

This means that the *m*-th order anti-stokes signal is also negatively chirped. When the negative chirp of the anti-Stokes sidebands just compensates the dispersion of the transparent bulk media and phase change in the medium, nearly-transform-limited pulses will be achieved.

#### 3.4.2. Generation of 15-fs multicolored femtosecond pulses

In this experiment, the pulse duration of beam2 was 75 fs with positive chirp, while that of beam1 was 45 fs with negative chirp. The filter used in the optical lay of beam1 was a SPF filter with cut-off wavelength at 800 nm. The spectra of the two input beams can refer to Figure 3. Their diameters on the surface of the glass plate were 250 μm (300 μm) in the vertical direction and 300 μm (600 μm) in the horizontal direction for beam1 (beam2). A 1-mm-thick fused silica plate was used as the nonlinear medium.

**Figure 12.**
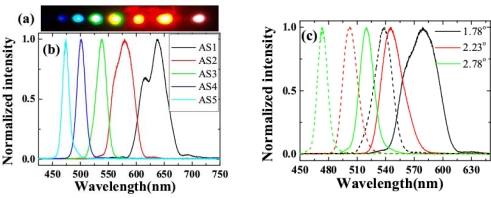
(a) Photograph of the sidebands on a sheet of white paper placed 30 cm after the glass plate when the crossing angle between the two input beams is 1.78°. The first, second, and third spots from the right-hand edge are beam2, beam1, and AS1, respectively. (b) Spectra of the sidebands from AS1 through AS5 when the crossing angle between the two input beams is 1.78°. (c) Spectra of AS2 and AS3 at crossing angles of 1.78°, 2.23°, and 2.78° [32].

Multicolor sidebands appeared discretely in space next to the two input beams, as shown in Figure 12(a). The photograph was the sidebands viewed on a white sheet of paper placed about 30 cm after the glass plate when the input powers of beam1 and beam2 were 15 and 24 mW, respectively. Figure 12(b) plots the spectra of the CFWM sidebands from AS1 through AS5 when the crossing angle was 1.78°. As expected, the bandwidths of the sidebands were wider than those of the sidebands obtained when the two input beams were slightly positively chirped. The transform-limited pulse durations of the sidebands were 9, 12, 16, 16, and 16 fs from AS1 through AS5, respectively. These pulse durations were much shorter than the value of ∼25 fs obtained in above sections when the two input beams had only a small positive chirp. Figure 12(c) shows the spectra of AS2 and AS3 at 1.78°, 2.23°, and 2.78° crossing angles. The spectra of AS2 and AS3 are both simultaneously tunable by changing the crossing angle. The tuning spectral range increased with increasing the order number from AS1 to AS5. The spectrum of AS3 at 1.78° is located between the spectra of AS2 when the crossing angles are 2.23° and 2.78°, indicating that the spectra of the sidebands are continuously tunable.

**Figure 13.**
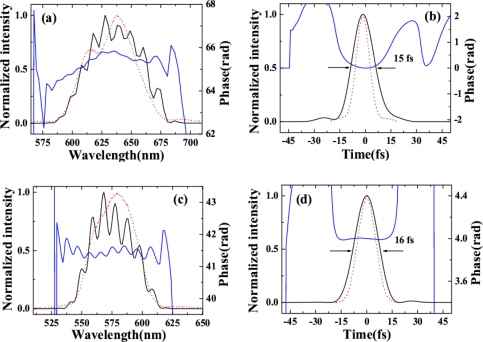
(a) Recovered spectrum (black solid curve), spectral phase (blue solid curve), and measured spectrum (red dotted curve) of AS1. (b) Recovered intensity profile and phase of AS1. The red dotted curve is the transform-limited (TL) (9 fs) pulse profile of AS1. (c) Recovered spectrum (black solid curve), spectral phase (blue solid curve), and measured spectrum (red dotted curve) of AS2. (d) Recovered pulse profile and phase of AS2. The red dotted curve is the TL (12 fs) pulse profile of AS1 [32].

The pulse durations of AS1 and AS2 were measured using XFROG. When there was no dispersion compensation, the XFROG trace of AS1 showed pronounced negative chirp. A 1-mm thick CaF_2_ plate was used to compensate the dispersion of the sidebands. The recovered intensity profiles, spectra, and phases of AS1 and AS2 were depicted in Figure 13 (a) to (d) with both retrieval errors of 0.014. Figure 13(a) graphs the measured and retrieved spectra of AS1 when the crossing angle is 1.78°. The measured and retrieved spectra agree well. The spectral phase indicates the presence of a small negative chirp in the pulse. The retrieved pulse profile and temporal phase are shown in Figure 13(b). The retrieved pulse duration is 15 fs, and its transform-limited pulse duration is 9 fs. A shorter pulse is expected when the negative chirp was completely compensated. The measured and retrieved spectra for AS2 are shown in Figure 13(c). The spectral phase of AS2 is nearly constant, except for some high-order dispersion. Figure 13(d) plots the retrieved pulse profile and the temporal phase, with the latter constant over the profile. The pulse duration is 16 fs, and its transform-limited pulse duration is 12 fs. AS1 has a small negative chirp while AS2 is nearly transform-limited because AS2 is a higher order CFWM sideband than is AS1. Therefore, the induced phase change of AS2 is larger and its negative chirp is smaller. Furthermore, the wavelength of AS2 is shorter than that of AS1. As a result, a 1-mm-thick CaF_2_ plate has the appropriate positive chirp to compensate the negative chirp of AS2. The resulting pulse duration was shorter than the obtained 35-fs sideband when the input pulses were positively chirped in Section 3.2.1. The output power of AS1 and AS2 are 0.65 and 0.15 mW, respectively.

#### 3.4.3. Generation of self-compressed ∼20-fs pulses

**Figure 14.**
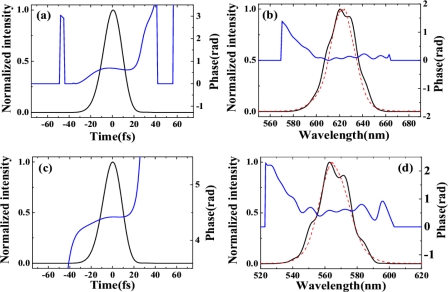
Recovered intensity profile and phase of (a) AS1 and (c) AS2. Recovered spectrum (black solid curve), spectral phase (blue solid curve), and measured spectrum (red dotted curve) of (b) AS1 and (d) AS2 [33].

Here, the input laser parameters were the same as Section 3.2.2. The pulse durations of AS1 and AS2 were measured by XFROG. The spectral and temporal profile and the phase were retrieved with a 256 × 256 grid. The retrieval error was smaller than 0.010. Figures 14(a) and 14(c) show the temporal profiles and phases of AS1 and AS2. The pulse durations of AS1 and AS2 are 20 and 22 fs, respectively, which are very close to the transform limited pulse durations of AS1 and AS2 (18 and 19 fs, respectively). The nonlinear phase changes due to crossing phase modulation and self-phase modulation, *etc.* in the medium combine with the dispersion of the 1-mm-thick glass compensate the chirps of the generated sidebands. The obtained pulse durations were even shorter than the transform-limited pulse durations of the two input beams, which are 32 fs and 33 fs for beam1 and beam2, respectively. Figures 14(b) and 14(d) show the retrieved spectra and the spectral phases of AS1 and AS2, respectively. The retrieved spectra reproduced the measured spectra very well. The retrieved phase shows a small positive chirp and some high-order dispersion. No dispersion compensation in the optical component for the output sideband was used in this process. In the process, the 1-mm-thick glass plate can only induce limited dispersion that can compress the pulse for about 1 fs in this wavelength. Then, the group velocity delay between two input pulses and the nonlinear phase in the process may affect the compression result. More detail analysis will be done through numerical simulation in the future.

### 3.5. Spatial Profile and Power Stability of Multicolored Sidebands

In many experiments, the spatial profile, beam quality, and power stability are very important. In our series of experiments, the spatial modes of the sidebands were measured using a CCD camera. All of the sidebands have Gaussian spatial profiles and good beam quality, even though the input beams have elliptical cross sections.

**Figure 15.**
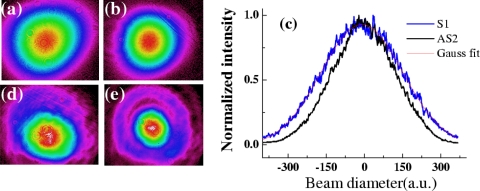
The two-dimensional spatial modes of (a) S1 and (b) AS3. (c) The one-dimensional spatial profiles of S1 and AS3, together with a Gaussian fit to S1. The two-dimensional spatial mode of beam2 (d) when beam1 was blocked and (e) when the input power of beam1 was 20 mW [31].

Following the experiment of Section 3.3, Figures 15(a) and 15(b) plot the two-dimensional spatial mode structure of S1 and AS3, respectively. Figure 15(c) shows the one-dimension spatial profiles of S1 and AS3. A Gaussian fit curve to S1 is also shown in Figure 15(c) and matches it well. The spot size of AS1 was first collimated using a concave mirror and then focused using a lens having a 700-mm focal length. The data of the diameter of the focal spot showed less than 1.1 times the diffraction limit. Weak diffraction-like rings around AS1 and AS2 were observed on a white screen. Interestingly, beam2 was focused and its spatial mode changed from elliptic to circular in cross section during the process, accompanied by diffraction-like rings. Figures 15(d) and 15(e) show the two-dimensional spatial mode of beam2 when beam1 was blocked and when the input power of beam1 was 20 mW, respectively. The resulting spatial mode improvement may be due to the generation of multicolor solitons [55,56].

**Figure 16.**
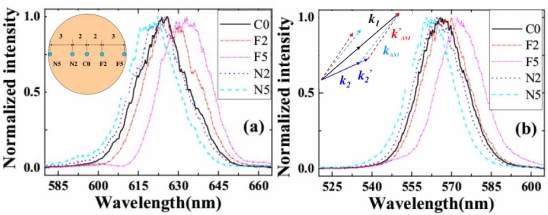
The inset pattern in Figure (a) shows the five different positions on the beam we measured. (a) and (b) show the spectra at the five different positions on the AS1 and AS2 sidebands, respectively. The inset of Figure (b) shows the phase matching geometry, which indicates longer wavelength on the side far from the two input beams [33].

In the experiment of Section 3.4.3, we measured the spectra of AS1 and AS2 at five different positions on the sidebands, as shown in the inset of Figure 16(a). The beam diameter was expanded to about 11 mm after propagating about 1.5 m in the air. C0 was the center measurement position. N2 and N5 were the two positions 2 mm and 5 mm, respectively, away from C0 on the side close to the input beams. F2 and F5 were the two positions on the side far from the input beams. Figures 16(a) and 16(b) demonstrated the normalized spectra profile at N5, N2, C0, F2 and F5 five different positions on the AS1 and AS2 sidebands, respectively. Longer wavelength located on the position far away from the two input beams while the shorter wavelength generated on the position close to the two input beams. The center wavelength was shifted for about 15 nm between the two edge points F5 and N5. It indicated that there was small angular dispersion in the sidebands. The angular dispersion can be explained by the different angles for phase matching condition to be deflected upon transmission from the crystal to air, as shown in inset of Figure 16(b).

**Figure 17.**
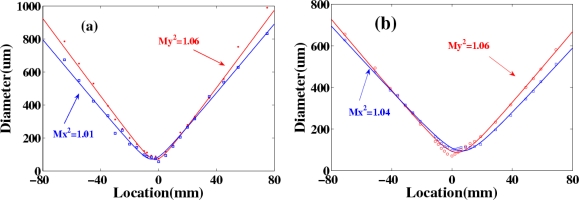
Measured beam diameters and M^2^ in both X and Y directions for (a) AS1 and (b) AS2 sideband, respectively [33].

The M^2^ values of AS1 and AS2 in this experiment were measured using a CCD camera to measure the beam diameters at more than 10 different positions on both sides of the focal point. The measured beam diameter data were fit by a hyperbola and obtained the value of M^2^ factor [57,58]. Figure 17(a) shows the obtained M^2^ value of AS1 at X and Y directions were about 1.01 and 1.06, respectively. Figure 17(b) shows that the M^2^ value of AS2 at X and Y directions were about 1.04 and 1.06, respectively. Both AS1 and AS2 owed perfect beam quality. These good beam qualities owe to the soliton effect in the mixing process [55,56].

**Figure 18.**
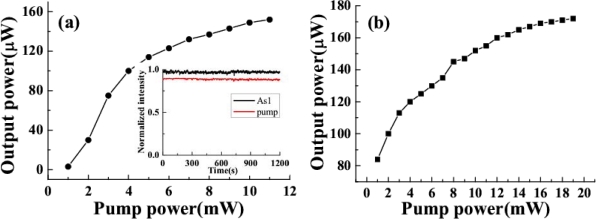
The dependence of the output power of AS1 on the power of one of the two input beams (a) dependence of beam1 power with the fixed power of beam2 of 19 mW; (b) dependence of beam2 power with the fixed power of beam1 of 11 mW. The inset in Figure (a) shows the power stability of AS1 and beam1 for twenty minutes [30].

The output power of the sidebands was monitored by a silicon sensor power meter in the experiment of Section 3.2.1. The dependence of the power of AS1 on the power of the two input beams was measured. Figure 18(a) shows the power of AS1 dependent on the power of beam1, which was used as the pump beam in this CFWM process while the power of beam2 was 19 mW and the crossing angle was 1.8°. The output power of AS1 was sensitive to the pump power. The output power of AS1 was found to be close to saturation as the pump power was increased to about 11mW. In this case, the power of beam1 was 11 mW and the crossing angle was 1.5°, and the AS1 output power was not very sensitive to the input power of beam2. However, the output power of AS1 became saturated as the input seed power increased. This saturation makes it possible to obtain sidebands with good power stability. The stability of the output power in terms of standard deviation of AS1 and beam1 was 0.95% RMS and 0.62% RMS, respectively, which was monitored at the same time for twenty minutes, as shown in the inset in Figure 18(a).

### 3.6. Generation of Multicolored Sidebands in BBO Crystal and Sapphire plate

As described in the introduction, multicolored sidebands can be observed in many kinds of bulk media. Here, we also show the results that multicolored sidebands generated in a BBO crystal and a sapphire plate. In the BBO crystal, the energy transfer efficiency was higher than that in the glass may owe to the different-frequency resonant in the media. In the sapphire plate, we observed more than ten columns and ten rows two-dimension multicolored arrays for the first time.

#### 3.6.1. Multicolored sidebands generated in BBO

**Figure 19.**
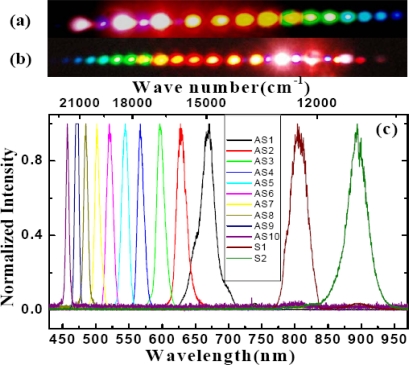
Photograph of the sidebands in a white sheet of paper behind the BBO crystal when (a) beam1 works as a pump. (b) beam2 works as a pump. (c) Spectra of sidebands from Figure (a), AS*m*(*m* = 1 – 10) refers to the *m*th-order anti-Stokes spectrum, S*n*(*n* = 1, 2) refers to the *m*th-order Stokes spectrum [46].

In the experiment, the pulse after chirped mirror was positively chirped to about 100 fs. No spectral filter was used for beam1 in this experiment. At first the crystal was placed before the focal point. The beam diameter on the crystal was about 0.8 mm for both beams. The pulses energy on the BBO crystal was 40 μJ and 54 μJ for beam2 and beam1, respectively. The two beams crossed on the crystal at an angle with each other of 1.75° in the air and nearly normal to the surface of the crystal. When the two input lasers beams temporally and spatially overlapped well, multiple brightness sidebands were generated beside the two input beams. A photograph of sidebands was taken on a white sheet of paper placed behind the BBO crystal, as shown on the top of Figure 19(a). The weak signals between the two input pulses and between the input pulse and the first order AS1 or S1 are the second harmonic signal and sum frequency mixing signal, as shown in the photograph in Figure 19. As many as fifteen AS sidebands and two S sidebands were generated. The spectra of different order sidebands were also measured and shown in Figure 19(c). The spectra of the sidebands can extend from the ultraviolet (421 nm) to the near infrared (980 nm) with more than one octave. When the delay of beam2 was slightly changed, multiple sidebands changed to the side of beam2 (Figure 19(b)). In this case, the pulse of beam2 was being used as a pump. The wavelength of the same order sidebands was different in Figure 19(a) and Figure 19(b). The frequency spacing between two neighbor sidebands was also different between Figure 19(a) and Figure 19(b).

**Figure 20.**
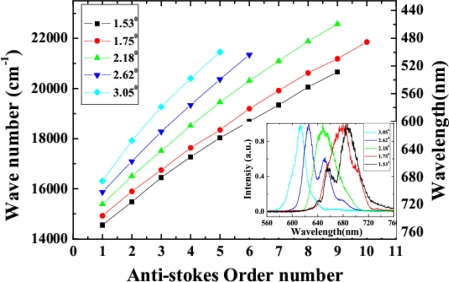
The center spectrum (wavenumber) of different sidebands varies with the anti-Stokes order number when the crossing angle between two input beams is 1.53°, 1.75°, 2.18°, 2.62° and 3.05°. The inset is the spectrum of the first order sidebands when the crossing angle between the two beams is 1.53°, 1.75°, 2.18°, 2.62°, and 3.05° [46].

Variability of spectra of the sidebands on the crossing angle was studied. In the experiment, two beams were set at five different crossing angles in the air, 1.53°, 1.75°, 2.18°, 2.62° and 3.05°. We found that the spectra of the sidebands and the conversion efficiency were changed by varying the crossing angle, as shown in Figure 20 and its inset. Brightest sidebands signals were observed when the crossing angle was 1.75°. As the crossing angle was decreased, the sidebands became close to each other both in frequency and space. Weak continuous line was generated when the crossing angle was reduced to smaller than 1.0°. The frequency space between the two different sidebands was increased gradually with the increase of the crossing angle between the two input beams. For example, the frequency space between AS1 and AS2 was increased from 924 cm^−1^ to almost 1,608 cm^−1^ when the crossing angle was increased from 1.53° to 3.05°, as shown in Figure 20. The spectrum of AS1 was shifted to high frequency with the increasing of the crossing angle. We could see that the center wavelength of the sidebands can be tuned in a large bandwidth by changing the crossing angle. As the sidebands order was increased, the frequency separateness between the neighboring sidebands was gradually decreased. The frequency space between AS1 and AS2 is about 1,117 cm^−1^, which is gradually decreasing to 691 cm^−1^ for AS8 and AS9.

**Figure 21.**
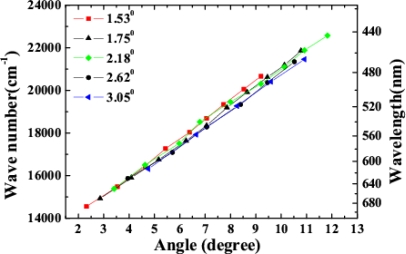
Relationship between the center wavenumber (wavelength) of different sidebands and the emitting angle of different sidebands. The crossing angle between the two input beams of which direction is normal to the crystal surface is 1.53°, 1.75°, 2.18°, 2.62°, and 3.05° [46].

The positions of different sidebands were also recorded by reading the stage position behind the BBO crystal. The distance between the crystal and the stage was 21 cm. In this way, the emitting angles for different sidebands were recorded. The dependence of the center wavelength and wavenumber of different order AS on the emitting angles at different crossing angles was plotted, as shown in Figure 21. As we can see, the slope for center wavenumber to output angle is almost constant, about 843 cm^−1^/degree, for BBO at different crossing angles. This means that the emitting angle of sidebands is correlated with the center wavelength of the sidebands not the order number. This linear relationship between the emitting angle and the wavelength of sidebands makes it possible to synthesize these sidebands by using dispersion optics that has been realized very recently by E. Matsubara *et al.* [38,42].

When the crystal was located on the focal point, the beam diameter was about 200 μm. When the energy of the input laser pulses was 3 μJ, bright sidebands were also observed in the experiment. High conversion efficiency larger than 30% is obtained when the crossing angle around 2 degrees. This is because the frequency difference between the two incident laser pulses in the calculation is close to the broad Raman line at 1.547 cm^−1^ [59]. As a result, the efficiency is enhanced by the difference frequency resonance.

#### 3.6.2. Multicolored sidebands generated in sapphire plate

**Figure 22.**
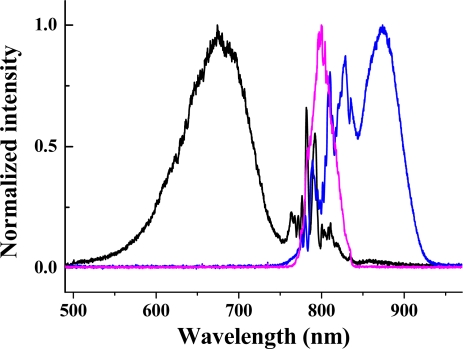
The spectra of beam2 (magenta line) and beam1 with an LPF with cut-off wavelength around 850 nm (blue line) or a SPF with the cut-off wavelength at 820 nm (black line) [34].

In the experiment, the beam diameters of beam1 and beam2 on the sapphire plate were about 200 and 800 μm, respectively. The crossing angle between beam1 and beam2 on the sapphire plate was 2.0°. At first, a 3-mm-thick LPF (R850) with cut-off wavelength around 850 nm was used to cut off the high frequency components of the beam1. The laser spectrum of beam1 after the R850 glass filter is shown in Figure 22 as blue solid line. The spectrum extends from 800 to 920 nm. The magenta color solid line shows the spectrum of beam2 with about 30 nm FWHM spectral bandwidth. The pulse energy after the R850 was 9 μJ and the pulse energy of beam2 was 195 μJ. Bright sidebands appeared at the side of beam2 in this case. Photograph of sidebands light was taken on a white sheet of paper, which was placed behind the sapphire plate.

**Figure 23.**
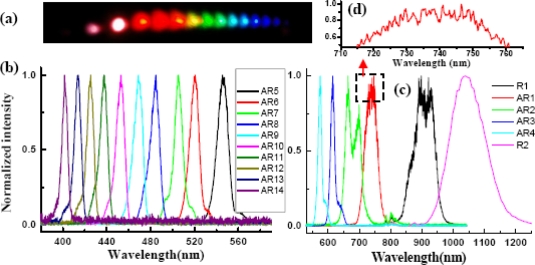
(a) Photograph and (b)spectra of the high-order sidebands and (c) the low-order sidebands (d) enlarged spectrum of peak-range of AR1 observed when a 3mm-thick R850 glass filter was used in beam1 path. AR*m* (*m* = 1 – 14) refers to the *m*th-order upshifted spectrum, and R*n* (*n* = 1, 2) refers to the *n*th-order downshifted spectrum [34].

Figure 23(a) shows the photograph of the sideband signals. As many as 15 upshifted sidebands and two downshifted sidebands were generated. The spectra of the sidebands extended from UV to near infrared with more than 1.5 octaves, as shown in Figures 23(b) and 23(c). To make it clearer, the spectra are shown in two figures. The second-order downshifted spectrum (R2) in near infrared was measured by an infrared spectrometer (NIR256-2.5, Ocean Optics). The spectral bandwidths of the low order upshifted and downshifted sidebands were even broader than that of the pump beam (beam2) owing to the broad spectrum of the two incident beams. The spectrum of the low order sidebands also exhibits an obvious clear interference pattern. An enlarged top part of AR1 spectrum showed in Figure 23(d). The same as that in BBO crystal described in Section 3.6.1, the frequency separation between two neighboring sidebands decreased from 1,203 cm^−1^ (AR1 and AR2) to 625 cm^−1^ (AR14 and AR13) as the order increased. The brightness and the number of sidebands were reduced when the pulse energy of beam1 or beam2 decreased.

**Figure 24.**
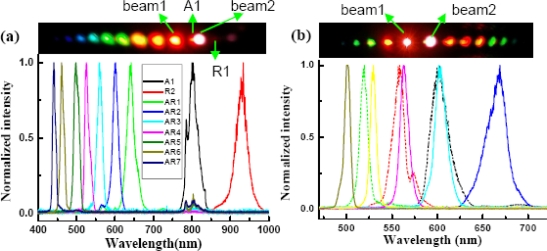
Photographs and spectra of the sidebands light (a) when a short-wavelength-pass glass filter with a cut wavelength at 820 nm was used in beam1 path; (b) when no glass filter was used in beam1 path. AR*m* (*m* = 1 – 7) refers to the *m*th-order upshifted spectrum, and R*n* (*n* = 2) refers to the *n*th-order downshifted spectrum [34].

In order to study the short wavelength range of the laser spectrum we replaced the R850 glass filter with another SPF filter with a cut-off wavelength at 820 nm. The pulse spectrum after the filter is also shown in Figure 22 with a black line. The spectrum extends from 600 to 820 nm. The pulse energy after the filter was about 40 μJ. The pulse energy of beam2 was about 115 μJ. In this case, bright sideband lights appeared only on the side of beam1 even the intensity of beam2 on the sapphire plate was higher than that of beam1. Photographs of sidebands light on a white sheet of paper placed behind the sapphire plate were shown at the top of Figure 24(a). As in Section 3.6.1, it was found that bright sidebands always appeared on the side of the beam with high frequency. Larger number of upshifted sidebands appeared than those of downshifted sidebands. There was a bright beam A1 between beam1 and beam2, which may seem strange at first sight. As we can see, the sideband overlapped slightly with beam2 in the spectrum and in space, as shown in Figure 24(a). This phenomenon is explained in terms of a cascade FWM signal between beam1 and AR1. In the process, the first order FWM signal AR1 was generated by beam1 and beam2, and then cascaded FWM process between beam1 and AR1 will be generated an upshifted beam AR2 and a downshifted beam A1. Both the spectrum and space shift are due to the phase matching condition.

The spectrum of the sidebands almost extends to the same spectral range resulting in more than 1 octave. The spectrum and direction from the sapphire plate of the sideband were dependent on the crossing angle between beam1 and beam2. When the crossing angle was smaller, the spatial gap and the spectral difference between neighboring sidebands were narrower. The intensity of the sidebands has optimum crossing angle. In the experiment, when the crossing angle was about 2°, the sidebands were brightest. The conversion efficiency from the pump energy to the sum of the sideband energies was about 12%. We also changed the polarization of beam2 normal to beam1, very weak few sidebands were observed.

In the experiment, when there was no filter inserted in the beam1 path, the spectrum of beam1 extends from 600 to 920 nm. Super-continuum light will be generated in this case due to high intensity on the sapphire plate. Therefore, we replaced the concave mirror to let the beam diameter of beam1 on the sapphire plate to be about 800 μm, which fits to the diameter of beam2. The pulse energy of beam1 was about 55 μJ. When the input pulse energy of beam2 is increased to about 195 μJ, bright up-shifted sidebands appeared on the both sides of beam1 and beam2 at the same time. In the experiment, to make sidebands to be separated clearly, the crossing angle between beam1 and beam2 on the sapphire plate was set at 2.75°. The photograph of the sidebands light on a white sheet of paper placed behind the sapphire plate was shown at the top of Figure 24(b). The spectra of the sidebands are shown as in Figure 24(b). The dotted lines and solid lines show spectra of sidebands on the sides of beam1 and beam2, respectively. The wavelengths of the sidebands of the same order on both sides were different from each other. Even though the spectra of the first-order signal and the second-order signal with cyan solid line are nearly overlapping, the spectra of high order were shifted to each other as the order increases. The spectral bandwidth of the sidebands with dotted line was a little broader than that of the near sidebands with solid line. Since the spectrum of the sideband was dependent on the crossing angle, there is a possibility to obtain larger number of sidebands by optimizing the crossing angle between beam1 and beam2.

**Figure 25.**
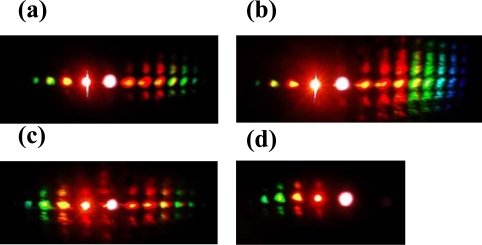
Photographs of the multicolored arrays under several different conditions. The conditions were, (a) pulse energy of beam2 was 220 μJ; (b) pulse energy of beam2 was increased to 250 μJ; (c) the delay of beam2 was tuned about 7 fs at 250 μJ input pulse energy; and (d) a short wavelength pass glass filter cut at 820 nm was inserted in the beam1 path [34].

Then, we increased the pulse energy of the beam2 by rotating the VND filter in beam2 path. As the pulse energy was increased to 220 μJ, 2-D multicolored arrays were observed clearly in the experiment, as shown in Figure 25(a). The 2-D multicolored arrays appeared only on the right of input beams. These 2-D arrays signals are found and show a periodic two-dimensional slightly deformed lattice structure. When the pulse energy of beam2 was increased further to 250 μJ, much more lines and brighter multicolored arrays were observed, as shown in Figure 25(b). The multicolored arrays only existed on the side of beam2. This may be due to the fact the two input beams are not absolutely normal to the sapphire plate that the multicolored arrays were slightly asymmetrical. As we tuned the delay of beam2 about 7 fs, multicolored arrays appeared on the both sides of beam1 and beam2. Figure 25(c) shows the photograph of the multicolored arrays on the both sides of beam1 and beam2. This is because that there were a lens and a VND filter in the beam2, which will induce positive chirp to the beam2. Then, a SPF cutting at 820 nm was inserted in the path of beam1, multicolored arrays again were observed, as shown in Figure 25(d). By varying the intensity, delay or polarization of one input beam, the 2-D multicolored arrays can be controlled.

### 3.7. 2-D Multicolored Sidebands Arrays Generated in Sapphire plate

**Figure 26.**
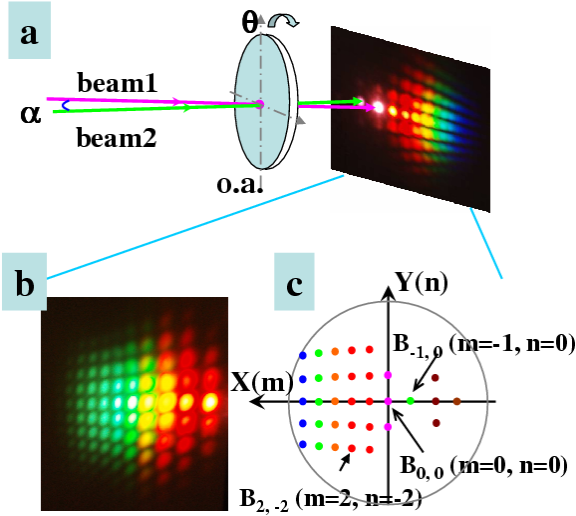
(a) Schematics of the experimental setup for multicolored array generation. α is the crossing angle between the two input beams, beam1 and beam2 in the air. θ is the rotation angle of the sapphire plate. (b) A photograph of the 2-D multicolored array on a UV light sensitive plate. (c) Definition of 2-D multicolored arrays, where B_0, 0_ and B_−1, 0_ refer to two incident beams, beam1 and beam2, respectively [35].

To study the 2-D multicolored sidebands arrays further in more detail, another experiment was performed. The schematic of the experimental setup for multicolored array generation is shown in Figure 26(a). The sapphire plate was a-cut and there were two orthogonal optical axes in the plane of the sapphire plate. The plane formed by the two orthogonal crystal axes was set normal to the two input beams. The two input beams had perpendicular polarizations and coincided with one of the crystal axes. The diameters of both incident beams on the surface of sapphire plate were 300 μm, as initially measured by a CCD camera. The crossing angle α between the two input beams was 1.80° ± 0.02° in the air. When beam1 and beam2 were focused on the sapphire plate synchronously in time and overlapped in space, stable separate 2-D transverse multicolored array signals at different wavelengths were generated. The polarizations of the multicolored arrays were the same as those of the input beams as tested using a film polarizer. Figure 26(b) shows a photograph of the 2-D multicolored arrays on a UV light sensitive plate placed about 20 cm after the sapphire plate. More than ten quasi-periodic columns and more than ten rows of multicolor signals can be seen, well separated from each other in space. The columns approximately normal to the center row and the rows adjacent to the center row were not parallel to the center row. For convenience, the 2-D multicolored array signals were defined to be B_m, n_, as shown in Figure 26(c), where B_0, 0_ and B_−1, 0_ refer to two incident beams, beam1 and beam2, respectively. There were also two sidebands, B_−2, 1_ and B_−2, −1_, beside the first-order Stokes sideband B_−2, 0_. The divergence angle of the sidebands was measured using a paper 50 cm after the sapphire plate to mark the position of each sideband. Neighboring spots on the same column have nearly the same crossing angle. However, the angle between two neighboring signals was decreased from 2.2° to 0.7° in the x direction (row direction, Figure 26(c)) and from 1.7° to 1.0° in the y direction (column direction, Figure 26(c)) as the sidebands changed from column B_−2, n_ to the B_7, n_ column.

**Figure 27.**
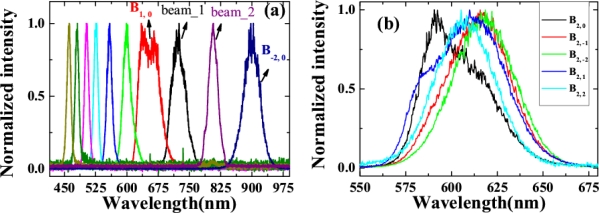
(a) The spectra of array signals on the center row B_m, 0_, where beam1 and beam2 are two incident beams. (b) The spectra of array signals on the second column B_2, n_ [35].

The spectra of array signals on the center row B_m, 0_ were measured using a multichannel spectrometer (USB4000, Ocean Optics), as shown in Figure 27(a), in which not all sideband spectra are shown for clarity. A broadband spectrum from 400 nm to 1.2 μm with more than 1.5 octaves can be generated. These generated center sidebands were explained to be the result of a CFWM process, which was the same as discussed in the previous Sections. The spectra were tunable by changing the crossing angle α between the two input beams, and also by changing the center wavelength of the BPF filter. The maximum difference in the wavelength between the side spots and the center spot on the same column was about 20 nm, as shown in Figure 27 (b).

**Figure 28.**
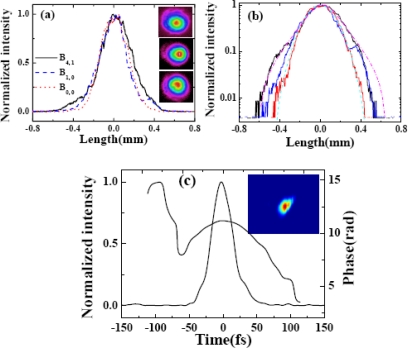
(a) The spatial profiles of B_0, 0_, B_1, 0_, and B_4, 1_ in one dimension. The inset patterns are spatial modals of B_0, 0_, B_1, 0_, and B_4, 1_ from bottom to top, measured by a CCD camera. (b) The spatial profiles of B_0, 0_, B_1, 0_, and B_4, 1_ in one dimension with logarithmic scale in the intensity. The cyan dashed line is the Gaussian fit of B_0, 0_. The magenta dash-dotted line is the Lorentzian fit of B_4, 1._ (c) The retrieved XFROG pulse trace and phase of the B_1, 0_ with a retrieved error of 0.01022. The retrieved pulse duration is 35 ± 3fs. The inset pattern is the measured XFROG trace [35].

The spatial profiles of different signals in the arrays were measured using a CCD camera. Figure 28(a) shows spatial profiles of B_0, 0_, B_1, 0_, and B_4, 1_ in two dimensions and one dimension. Figure 28(b) shows one-dimensional spatial profiles of B_0, 0_, B_1, 0_, and B_4, 1_ with logarithmic scale in the intensity. The spatial profile changed from Gaussian (B_0, 0_) to a Lorentzian profiles (B_4, 1_). The cyan dashed line in Figure 28(b) is the Gaussian fit of B_0, 0_. The magenta dash-dotted line in Figure 28(b) is the Lorentzian fit of B_4,1._ The pulse duration of the sidebands were measured by cross-correlation with beam4. Figure 28(c) shows the retrieved XFROG pulse trace and the phase of B_1, 0_ with a retrieved error of 0.010. The retrieved pulse duration was 35 ± 3 fs. The retrieved phase shows that there was some chirp in the pulse due to the positive chirped input pulses and the dispersion of the glass.

**Figure 29.**
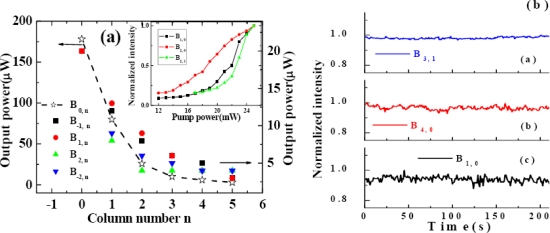
(a) The output power of array signals on the 0, ±1, and ±2 rows when the power of the two incident beams, beam1 and beam2, were 0.1mW and 25mW, respectively. Only the signals in the center row are marked with star symbols and dashed line, as shown on the left. The inset figure shows the dependence of the output power of different sidebands (B_1, 0_, B_2, 0_, and B_2, 1_ from top to bottom) on the input power of beam2. (b) The power stabilities of B_4, 0_, B_3, 1_, and B_1, 0_ monitored for 200 seconds, which were 1.25%RMS, 0.63%RMS, and 1.84% RMS, respectively [35].

We measured the powers of some array signals when the power of the two incident beams, beam1 and beam2, were 0.1 and 25 mW, respectively, as shown in Figure 29(a). The signal power of the center row B_m, 0_ decreased rapidly with increasing column order, as shown by the star symbols in Figure 29(a). The difference in output power between the side spots and the center spot on the same column decreased continuously as the row order increased. For signals on column 5 (B_5, n_), signal power on the center row B_5, 0_ was even smaller than that on rows ±1 and ±2. The dependence of the different sidebands, B_1, 0_, B_2, 0_, and B_2, 1_ from top to bottom, output power on the input power of beam2 is shown in the inset of Figure 29(a). In this case, the output power of beam1 was amplified from 0.1 to 0.17 mW in the experiment. The power stabilities of different array signals were monitored by a Si power sensor, as shown in Figure 29(b). The stability of B_1, 0_ was about 1.84% RMS over 200 seconds. Interestingly, the stabilities of the high order sidebands were much better than that of the first-order, especially for the sidebands beside the beam on the center row. The stabilities of B_4, 0_, B_3, 1_, and B_4, 1_ were 1.25%RMS, 0.63% RMS, and 0.97% RMS, respectively over 200 seconds.

**Figure 30.**
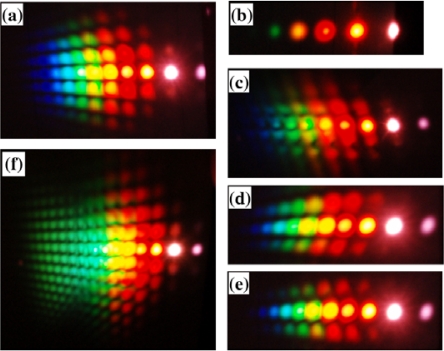
Photographs of 2-D multicolored arrays on a sheet of white paper when (a) the plane of polarization of the two input beams coincided with one of the crystal axes; (b) the sapphire plate rotated for 45°; (c) noise pattern; (d) and (e) show photographs with the sapphire plate rotated by −16° and 14°, respectively. (f) A photograph of the 2-D multicolored arrays on a UV light sensitive plate when the input power of beam2 was increased to 27mW [35].

We observed the multicolored arrays to be sensitive to the rotation of the sapphire plate in the plane normal to the input beams. The brightest and largest number of array signals appeared when the plane of the polarization of the two input beams coincided with one of the crystal axes, as shown in Figure 30(a). It need to clarify that the white color of the two input beams due to the over exposure of camera. There is no white light generation in the two input beams. It was found that there were four angles 0°, 90°, 180°, and 270° at which the sidebands were brightest, and four angles 45°, 135°, 225°, and 315° at which the sidebands were weakest, as shown in Figure 30(b). The periodic array did not appear continuously but appeared aperiodically *i.e.* when the angle was rotated 6°, 14°, 0°, −6°, −11°, −13°, −16°, and −19° and further. At other angles, these regular arrays were replaced by a noise pattern, as shown in Figure 30(c). The rotation angle of the sapphire plate also affected the position of the array signals. Figures 30(d) and 30(e) showed photographs when the sapphire plate was rotated by −16° and 14°, respectively. It can be seen that the column line was tilted a different direction when the sapphire plate was rotated. The spectrum of the tilted signal was slightly narrower in the shorter wavelength region when the signal was tilted in the high order direction, and vice versa. If the power of beam2 was increased to 27 mW, bright stable “fish-like” multicolor arrays were observed, as shown in Figure 30(f).

**Figure 31.**
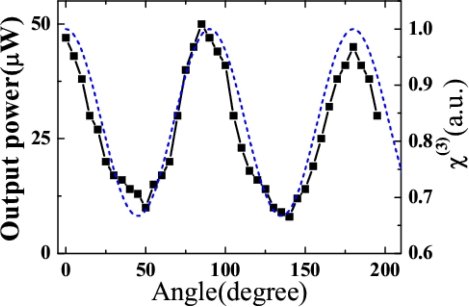
The dependence of B_1, 0_ output power (square symbols) and |χ^(3)^(θ)| (dashed line)of the sapphire plate on the rotation angle θ of the sapphire plate [35].

The dependence of the B_1, 0_ output power on the rotation angle θ of the sapphire plate was measured, as shown in Figure 31. Here we only show the evolution in the rotation angle region from 0° to 210°. Clearly, the output power changed periodically with rotation of the sapphire plate. This periodic evolution was because of the periodic dependence of χ^(3)^(θ) ∝ cos^2^(2θ) + 1of the sapphire plate on the rotation angle θ, as shown by the dashed line in Figure 31. The peak wavelength of the sidebands shifted continuously to a shorter wavelength by about 20 nm when the sapphire plate was rotated from 0° to 45° due to a phase matching condition. The polarization of the sidebands also changed with rotation of the sapphire plate. To detect the polarization rotation of the sidebands, a thin film polarizer was located normal to the sideband beams and placed 50 cm after the sapphire plate. The polarizations of the multicolored arrays were the same as those of the input beams when the plane of polarization of the input beams coincided with one of the crystal axes. Only very weak orthogonally polarized light was detected. As the sapphire plate was rotated from 0° to 90°, the orthogonally polarized light continuously increased from 0° to 45° and decreased from 45° to 90°, and light with parallel polarization showed the opposite effect. At 45°, the sidebands were the weakest, and the orthogonally polarized light had power equal to the parallel-polarized light. The thin film polarizer was rotated to minimize the intensity of light passing through the film polarizer. It was found that the rotation angle of the thin film polarizer β was in accordance with the rotation angle of the sapphire plate θ, and had the same angle when the sapphire plate was rotated from 0° to 45° and was 90° – θ when the sapphire plate was rotated from 45° to 90°. This rotation angle dependence was also recently observed in a supercontinuum generation process [60]. The physical explanation of the phenomenon is complex because it included combined effects of the cascaded third-order nonlinear processes of FWM, coherent anti-Stokes Raman scattering (CARS), XPM, and self-phase modulation. Much detail physical explanation of this phenomenon will be explored in the future.

A half-wave plate or a quartz-wave plate can also be used in the path of one of the input beams to safely control the multicolored arrays. The polarization, intensity, and position of the multicolored arrays can be controlled by rotating the sapphire plate. Note that this phenomenon did not appear when a fused silica glass was used as the medium, due to its symmetric structure. This phenomenon was very easily repeated in the experiment, and the sapphire plate was not damaged over the course of the experiment.

### 3.8. Simultaneous Compression and Amplification of the Sidebands in a Bulk Medium

In the present CFWM system, the pulse duration and output pulse energy of the obtained sidebands by using CFWM in bulk media were limited at sub-20 fs and 1-μJ level. In this section, we proposed and demonstrated for the first time a method for simultaneous compression and amplification of a weak femtosecond pulse in a bulk medium using XPM in conjunction with FWOPA [19,47–53] that is pumped by an intense femtosecond pulse.

#### 3.8.1. Principle of the experiment

**Figure 32.**
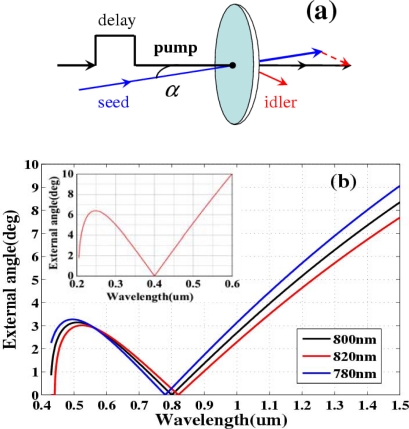
(a) Schematic of the experimental setup; *α* is the crossing angle. (b) Phase-matching curves showing the crossing angle *α* as a function of the seed wavelength for fused silica (or CaF2, inset) when the pump pulse was fixed at a typical wavelength of 780, 800, and 820 nm (or 400 nm, inset) [50].

The principle of this novel method is schematically illustrated in Figure 32(a). An intense pump beam and a weak seed beam are focused onto a glass plate with a crossing angle *α*. Usually, the wavelength of the pump pulse is fixed; it should be different from that of the seed pulse to prevent interference between the two pulses. When the pump and seed pulses are synchronous in time and overlapping in space in a transparent bulk medium, the seed pulse spectrum will be broadened due to the XPM effect in the medium induced by the intense pump pulse [61–65]. Furthermore, the weak seed pulse will be simultaneously amplified when the crossing angle *α* satisfies the phase-matching condition of FWM [19,47–53].

A diagram of the vectors of FWM is also presented in Figure 32(a). According to the phase-matching condition, the crossing angle *α_in_* in the medium can be represented by cos *α_in_* = [(2*k_p_*)^2^ + *k_s_*^2^ − *k_i_*^2^]/4*k_p_k_s_*, in terms of wavenumber *k* with the subscripts of *p*, *s*, *i* indicating the pump, seed, and idler beams, respectively. Figure 32(b) and its inset show the plots of the phase-matching curves of the crossing angle in air *α* as a function of the seed wavelength for fused silica and for CaF_2_, respectively. The pump pulse is fixed at a typical wavelength of 800 nm and 400 nm for fused silica and CaF_2_, respectively. As indicated in Figure 32(b) (or inset of Figure 32(b)), there is a broad phase-matching bandwidth around 500 nm (or 250 nm) when the crossing angle *α* is about 3.1° (or 6.4°).

#### 3.8.2. Experiment results and discussion

**Figure 33.**
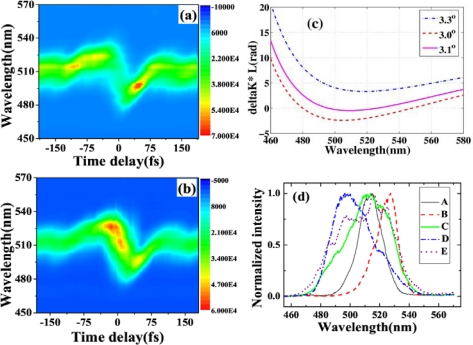
Dependence of the spectral profile and intensity of the output seed beam (AS1) on the delay time *t_ps_* for crossing angles *α* of (a) 1.80° ± 0.05° and (b) 3.30° ± 0.05°. (c) The phase mismatch curves at three different external crossing angles (3.3°, 3.1°, and 3.0°) in a 0.5mm-thick fused silica glass when the seed pulse was centered at 510 nm and pumped by 800 nm pulse. (d) A: the incident spectrum of seed pulse. B, C, and D are the spectra of output pulse at delay times of −30, 0, and 30 fs when *α* was 3.30° ± 0.05°, respectively. E: the spectra of output pulse at delay times of 0 fs when *α* was 1.80° ± 0.05° [50].

Initially, the third-order anti-Stokes (AS3) sideband was used as the seed beam because its center wavelength is close to the broad phase-matching spectral range around 500 nm. The incident pulse energies of the AS3 and pump beams were 300 nJ and 140 μJ, respectively. The spectral profile and spectral intensity of the output seed beam as a function of the delay time *t_ps_* when the crossing angle *α* was 1.80° ± 0.05° are shown in Figure 33(a). A negative delay time *t_ps_* indicates that the seed pulse precedes the pump pulse. The full width at half maximum (FWHM) spectral bandwidth of the seed pulse was smoothly broadened from 18 to 50 nm (*i.e.*, by a factor of about 2.8) at a delay time of approximately 0 fs due to XPM. This broadened spectrum can support transform-limited pulse duration of 9.6 fs. In this case, the spectral broadening of a Gaussian pulse due to XPM can be simply expressed as Δ*ω_s_* ≈ *ω_s_* / *cn*_2_(|*E_s_*|^2^ + |*E_p_*|^2^)*l*/*T*_0_ [62,65] because the intensity of the pump pulse is nearly undepleted. In the expression, *ω_s_* is the angle frequency of the seed pulse, *n_2_* is the nonlinear refractive index of the medium, *E_s_* and *E_p_* are electric field amplitudes of seed pulse and pump pulse, respectively, *l* is the thickness of bulk medium, *T_0_* is the FWHM pulse duration of the pump pulse. Using the above expression, the spectral broadening is calculated to be about 40 nm in agreement with the experimental result. In this case, the seed beam was not amplified due to the large phase mismatch. When the crossing angle *α* was 3.30° ± 0.05°, the FWM phase-matching condition was satisfied (Figure 32(b)). In this case, the spectrum of the seed pulse was smoothly broadened and its output power was simultaneously amplified, as shown in Figure 33(b). The pulse energy of the seed pulse was amplified from 300 to 940 nJ (*i.e.*, by a factor of about 3.1) at a delay time of approximately 0 fs. The spectrally asymmetric amplification in the Figure 3(b) is due to the phase matching condition satisfied in broader spectral range on the longer wavelength side, as shown in Figure 33(c). The phase-matching angle in the experiment (3.30° ± 0.05°) was slightly larger than the calculated value for the best phase matching as shown in Figure 33(c) and Figure 32(b).

This is because of the nonlinear phase shift φ*_NL_* = Δ*k*′*l* = 2*ω_p_n*_2_*I_p_l*/*c* induced by the nonlinear index [63,65]. In the expression, *ω_p_* and *I_p_* are the angle frequency and intensity of pump pulse, respectively. This induced additional phase amounts to be about 3*π* phase in a 0.5 mm fused silica glass under the experimental condition. The spectra of the output seed pulse at delay times of −30, 0, and 30 fs are shown in Figure 33(d).

**Figure 34.**
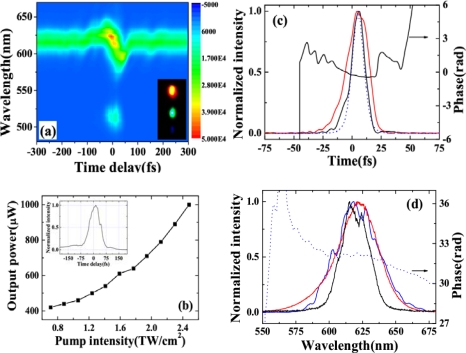
(a) The spectral profile and intensity of the output seed beam (AS3) as a function of the delay time *t_ps_* when the crossing angle *α* was 2.80° ± 0.05°. (b) The dependence of the output energy of the output seed pulse on the pump intensity. The inset shows the temporal profile of pump pulse. (c) The retrieved temporal profiles of the incident seed pulse (red solid line) and the compressed output seed pulse (black solid line), and the transform-limited pulse of the broadened spectrum (blue dotted line). (d) The retrieved spectrum (blue solid line) and the spectral phase (blue dotted line) of the output seed pulse. The measured spectra of the incident seed pulse (black solid line) and output seed pulse (red solid line) [50].

The seed pulse spectrum was clearly red-shifted (blue-shifted) when the two incident pulses were overlapping in negative (positive) delay time. The peak wavelength of the seed pulse can be shifted by about 20 nm on both sides, indicating that the spectrum of the amplified output pulse can be tuned by adjusting the delay time *t_ps_*. This spectral shift can be easily explained as follows. The frequency shift induced by XPM can be given by *δω*(*t*) = −∂φ*_NL_* / ∂*t* ∝ −∂|*E_p_*(*t*)|^2^ / ∂*t*, where *E_p_*(*t*) is the electric field of the pump pulse. It can be concluded from the above expression that *δω*(*t*) < 0 at the leading edge of the pump pulse and *δω*(*t*) > 0 at the trailing edge of the pump pulse. As for the negative delay time, the leading edge of the pump pulse will overlap with the seed pulse. Therefore, the induced frequency change is negative (*δω*(*t*) < 0) and the spectrum of the seed pulse is red-shifted; vice versa for a positive delay time. This phenomenon was observed also when the first-order anti-Stokes (AS1, 620 nm) sideband was used as the seed beam. When the crossing angle *α* was around 2.80° ± 0.05°, the incident seed pulse was spectrally broadened and amplified simultaneously. In this case, the incident pulse energy of AS1 was 400 nJ and the pump energy was 140 μJ. The spectral profile and intensity of the amplified output beam as a function of the delay time *t_ps_* are shown in Figure 34(a). CFWM signals were simultaneously generated in this case, as shown in the inset photograph of Figure 34(a).

The maximum output pulse energies of the seed beam and the first-order cascaded signal (around 500 nm) were 1.1 μJ and 250 nJ, respectively. The output energy of the seed pulse as a function of the pump intensity at a delay time of 0 fs is shown in Figure 34(b). Much higher output energies are expected to be obtained when cylindrical lens for focusing is used [47–53]. The small thickness of the glass plate ensured the broad enough spectral bandwidth of phase-matching, resulting in broadband amplification around 620 nm. In the process, the pump pulse has a slightly steeper trailing edge, which was measured by using SHG-FROG, as shown in the inset of Figure 34(b). Furthermore, the self-steepening effect will introduce a sharper trailing edge of the pump pulse during its propagation [65]. As a result, the rapid decrease of trailing edge induced a broader blue spectral shift in the seed pulse, as shown in Figure 33(a), 33(b) and Figure 34(a). The steep trailing edge of the pump pulse also induced a rapid decrease of the spectral shift in the positive delay time, as is also shown in Figure 33(a), 33(b) and Figure 34(a).

A quasi-linear chirp can be imposed across the weak seed pulse when the pump pulse is much wider compared with it [65]. In the same way as SPM-based compressors, the phase induced by XPM can also be compensated by using a chirped mirror pair. Furthermore, XPM based compressor can have more flexibility than SPM ones because the phase can be tuned by the pump pulse. After four bounces on a pair of chirped mirrors (−40 fs^2^/bounce), the pulse duration of the weak output pulse was measured by XFROG. The spectral and temporal profiles and the phase were retrieved with a 512 × 512 grid. The retrieval error was smaller than 0.006. The retrieved temporal profiles of both the incident seed pulse and the compressed output seed pulse, and the transform-limited pulse for the broadened seed pulse are presented in Figure 34(c). The 22.6 ± 0.5fs incident pulse was compressed to 12.6 ± 0.5 fs, which is well close to the calculated transform-limited pulse duration of 10.5 fs. The retrieved spectrum and spectral phase of the output seed pulse are shown in Figure 34(d).

**Figure 35.**
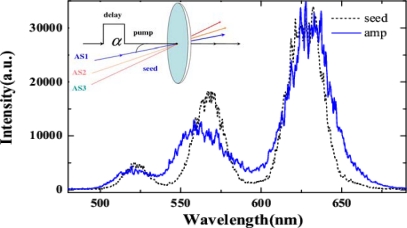
The spectral profiles of AS1, AS2, and AS3 without pump (black dash line). The blue solid line is the spectral profiles of AS1, AS2, and AS3 with pump. The inset is a schematic of the experimental setup; *α* is the crossing angle between pump and AS1 [50].

We also guided three beams, AS1, AS2, and AS3 generated by CFWM into the glass at the same time. The schematic of the experimental setup was shown in the inset of Figure 35. The beam diameters of AS1, AS2, and AS3 on the 0.5-mm thick fused silica glass were all about 190 μm. The crossing angles between pump beam and three anti-Stokes sidebands, AS1, AS2, and AS3 were about 2.6°, 2.9°, and 3.2°, respectively. The pump pulse energy was 160 μJ with about 350μm diameter on the glass. When these seed and pump beams synchronized in time on the glass, the spectra of the three seed beams, AS1, AS2, and AS3 were broadened simultaneously, as shown in Figure 35. The pulse energy of AS1, AS2, and AS3 were also amplified from 133, 40, and 12 nJ to 163, 50, and 15 nJ, respectively. This experiment shows that it is possible to simultaneously amplifying and compressing several weak pulses in a bulk medium at the same time. Then, it can be used for cascaded FWM process to optimize the spectrum of the obtained sidebands which was expected to obtain single cycle pulse after combine the optimized sidebands [28].

## 4. Theoretical Calculation of the CFWM Process

In the above Experiment section, we found that the frequency difference between two adjacent sidebands decreased with increasing order number. The following calculation explains this phenomenon semi-quantitatively. This is because that both input beams are femtosecond pulses and they thus have wide spectral bandwidths. As a result, a different spectral band was selected from the broad input spectra, which induced this variable frequency gap between two adjacent sidebands with increasing order number.

To simplify the calculation, we set the wavelength of one input beam to 800 nm (*ω*_2_) (beam2) and the wavelength of the other input beam to a wide bandwidth from 660 to 740 nm with a center wavelength at 700 nm (*ω*_1_) (beam1); this accords with the frequency parameters for the two input beams in our experiment above section 3.3. For a given external crossing angle in the same material, the frequencies and wave vectors of CFWM sidebands are determined by the phase-matching conditions: ***k*_AS*m*_** = ***k*_AS(*m−1*)_** + ***k^(m)^*_1_** − ***k^(m)^*_2_**, *ω_ASm_* = *ω*_*AS*(*m*−1)_ + *ω*_1_^(*m*)^ − *ω*_2_^(*m*)^ and ***k*_S*m*_** = ***k*_S(*m−1*)_** + ***k^(−m)^*_2_** − ***k^(−m)^*_1_**, *ω_Sm_* = *ω*_*S*(*m*−1)_ + *ω*_2_^(−*m*)^ − *ω*_1_^(−*m*)^ from section 2. We calculated this CFWM process step by step. In every step of FWM process, we scan the wavelength of *ω*_1_ from 660 to 740 nm to find the minimum phase mismatching **Δ*k*_AS*m*_** = ***k*_AS(*m−1*)_** + ***k^(m)^*_1_** − ***k^(m)^*_2_** − ***k*_AS*m*_** or **Δ*k*_S*m*_** = ***k*_S(*m−1*)_** + ***k^(−m)^_2_*** − ***k^(−m)^*_1_** − ***k*_S*m*_**. For example, from Figure 1(b), the phase mismatch **Δ*k*_AS1_** depends only on the wavelength of *ω*_1_ from 660 to 740 nm when all the other input parameters are fixed. Then, the output parameters, including direction and wavelength, of the first-order sideband (AS1) can be determined by finding the minimum phase mismatch **Δ*k*_AS1_** when scanning the wavelength of *ω*_1_ from 660 to 740 nm. After the parameters of AS1 have been determined, the output parameters of AS2 can be determined in the same way of finding the minimum phase mismatch **Δ*k*_AS2_** through scanning the wavelength of *ω*_1_ from 660 to 740 nm, as shown in Figure 1(c). It can be concluded that all of the output parameters of the sidebands can be determined by finding the minimum phase mismatch **Δ*k*_AS*m*_** or **Δ*k*_S*m*_**. In this way, we can calculate the exit angle and wavelength of the generated cascaded FWM sidebands, and the wavelength of *ω*_1_ at the minimum phase mismatch for every step of CFWM.

**Figure 36.**
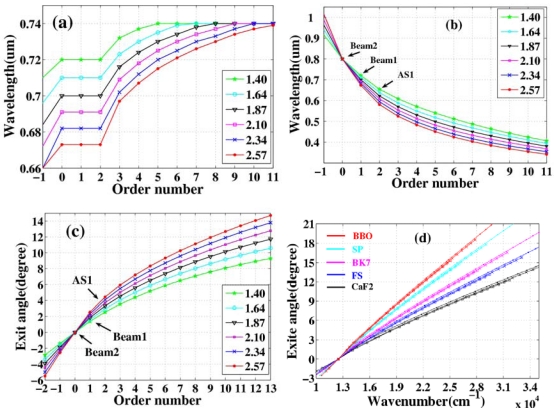
Order number dependence of (a) the wavelength of generated sidebands, (b) the wavelength of beam 1 for optimal phase matching, and (c) the exit angles of the generated sidebands obtained at six different crossing angles (1.40°, 1.64°, 1.87°, 2.10°, 2.34°, and 2.57°) in a 1-mm-thick fused silica plate. (d) The dependence of the exit angle of the generated sidebands on the center wavelength of generated sidebands at 1.40°, 1.87°, and 2.57° three different crossing angles in five different media (CaF_2_, fused silica, BK7, sapphire plate, and BBO crystal) [33].

Here, we calculated the output parameters of the generated sidebands when the material is a 1-mm-thick fused silica plate for several external crossing angles (1.40°, 1.64°, 1.87°, 2.10°, 2.34°, and 2.57°) which are the conditions in the experiment described in Section 3.3. Figure 36 shows the calculation results, where an order number of 0 refers to beam2 with 800 nm (*ω*_2_), an order number of 1 refers to the wavelength of beam1 between 660 to 740 nm (*ω*_1_) when the minimum phase mismatching **Δ*k*_AS1_** are obtained, and an order number of 2 refers to the first-order anti-Stokes sideband (AS1), *etc.* Figure 36(a) shows the wavelength of beam1 for generating different order sidebands in the case of the phase mismatching is the minimum. In Figure 36(a), the order numbers 0 and 1 refer to beam2 and beam1, respectively. The wavelength related to order number 2 refers to the wavelength of *ω*_1_ for generating AS1 in the case of the phase mismatching is the minimum, and so on. It can be seen that the optimal external crossing angle that the minimum phase mismatching for AS1 was obtained at the center wavelength of *ω*_1_ 700 nm in this case is about 1.87°, which is in good agreement with the experiment data. The wavelength of beam1 increases with the order number of the anti-Stokes sideband. This explains why the wavelength gap between two adjacent sidebands decreases with increasing in the order number. For the higher-order numbers, the frequency difference between two adjacent sidebands becomes equal. This is because the two input pulses have limited spectral bands. The wavelength of beam1 increases and fixed at the maximum wavelength of 740 nm in this calculation at higher-order numbers (see Figure 36(a)). For the same order number, the wavelength difference of *ω*_1_ induces also a different wavelength gap between two adjacent sidebands for different external crossing angles. We also can see that the wavelength of beam1 decreases for the same-order sideband with the increase of the external crossing angles. This is the reason that the spectra of the generated sidebands are tunable by changing the external crossing angles. It also can explain the evolution of output power of sidebands at different external crossing angle in the Figure 8(b) in section 3.3. It is because both input pulses have Gaussian spectral profile with the highest intensity of *ω*_1_ locating at a center wavelength of 700 nm and with decreasing intensities on both side directions. Then, the output power of the sideband will be higher when the wavelength of *ω*_1_ close to 700 nm and lower when it is far from 700nm. When the external crossing angle is 1.4°, the S1 and AS1 have high output power and decreasing quickly with the order number increasing. When the external crossing angle is 1.87°, the S1 and AS1 also have high output power. However, the output powers from AS2 to AS5 are higher and decreasing more slowly with the order number increasing compare with that at 1.4° crossing angle. These results are in agreement with wavelength of *ω*_1_ in Figure 36(a).

It can be explained in the same way when the external crossing angles are 2.10° and 2.57°. It explains that it is difficult to obtain higher order Stokes sideband. Figure 36(b) shows the evolution of the wavelength of the generated sidebands with the order number at several different external crossing angles (1.40°, 1.64°, 1.87°, 2.10°, 2.34°, and 2.57°). It clearly shows that the wavelength of the same order sideband can be tuned by changing the external crossing angle. Figure 36(c) shows the exit angles of different sidebands at several different external crossing angles, the evolution agrees well with the experiment data given in Figure 8(a) in section 3.3. Figure 36(d) shows the curve of the dependence of the exit angle of the generated sidebands on the center wavelength of generated sidebands for several different media. It is interesting to see that the curves are almost overlapped for different external crossing angles for the same medium. Here, in Figure 36(d), we only display the curves at 1.40°, 1.87°, and 2.57° three different angles. The slope of wavenumber to exit angle are about 830, 916, 1,136, 1,315, and 1,602 cm^−1^/degree for BBO crystal, sapphire plate, BK7, fused silica, and CaF_2_, respectively. As for BBO crystal, this result is in accordance with Figure 20 in Section 3.6.1.

**Figure 37.**
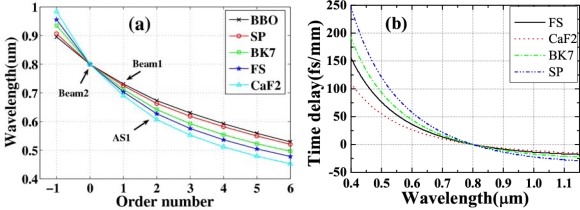
(a) Dependence of the wavelength of generated sidebands on the order number in five different media (CaF_2_, fused silica, BK7, sapphire plate, and BBO crystal) when the crossing angle is 1.8°. (b) The group delay between beam2 (800 nm) and other wavelength in CaF_2_, fused silica, BK7, and sapphire plate with 1mm thickness [33].

Figure 37(a) shows the dependence of the wavelength of every sideband on the order number at several different transparent bulk media (CaF_2_, fused silica, BK7, sapphire plate, and BBO crystal) when the crossing angle fixed at 1.8°. It is clearly seen that the wavelength of every sideband is tunable at a fixed external crossing angle in various transparent bulk media. This means that the center wavelength of the generated multicolor sideband can be tuned by simply replacing the nonlinear medium. We also calculated the group delay between beam2 (800 nm) and other wavelength in CaF_2_, fused silica, BK7, and sapphire plate, as shown in Figure 37(b). It can be concluded that for the purpose of obtaining broadband sidebands [27,28], it is advantageous to use a bulk medium with lower dispersion, for example CaF_2_ and fused silica, and use a thin (∼150 μm) glass as the nonlinear medium in the process to reduce time delay.

The above calculations are all based on the phase matching condition, which only indicates the important role of the phase matching in this process. Although it can qualitatively explain the process, this is inadequate when the two input pulses are femtosecond pulses with broadband spectrum. For a much more precise description and to understand the process fully, the analysis should begin with an integral description of the FWM nonlinear polarization density just the same as reference [54,66]. The third-order dielectric polarization induced at frequency Ω by the input beams can be expressed by summing over all possible permutations of the input frequencies according to the third-order susceptibility [66]:

(3.1) $$\begin{array}{ll} {\tilde{P}}^{\left(3\right)}\;(z,\;\Omega )= & \int \int d\omega_{1}d\omega_{2}\;{\tilde{\chi }}^{\left(3\right)}(\omega_{\mathrm{eg}}-\omega_{1},\;\omega_{1}-\omega_{2},\;-\omega_{\mathrm{eg}}+\Omega ){\tilde{E}}_{1}^{*}\;(z,\;\omega_{1}){\tilde{E}}_{2}(z,\;\omega_{2}){\tilde{E}}_{3}(z,\;\Omega -\omega_{2}+\omega_{1})\\ & \times \text{exp}[i(-k_{1z}(\omega_{1})+k_{2z}(\omega_{2})+k_{3z}\;(\Omega -\omega_{2}+\omega_{1}))z] \end{array}$$

In the expression, we assume the pulses are overlapped very well in time. Representation of the frequency-dependent third-order nonlinear susceptibility, *χ̃* ^(3)^(*ω_eg_* − *ω*_1_, *ω*_1_ − *ω*_2_, −*ω_eg_* + Ω), is based on the interaction of the input fields with an electronic transition with the frequency *ω_eg_*. In the case of a nonlinear process with a low efficiency (*E*_1,2,3_ = *const*), the four-wave mixing signal field can be obtained through integrate the signal intensity over the longitudinal coordinate z, as follows:

(3.2) $$\begin{array}{ll} {\tilde{E}}_{4}(L,\Omega ) & =i\frac{c\mu_{0}\Omega }{2n_{4}(\Omega )}\int_{0}^{L}{\tilde{P}}^{\left(3\right)}\;(z,\Omega )\;\text{exp}(-{\mathrm{ik}}_{4z}\;(\Omega )z)\mathrm{dz}\\ & =i\frac{c\mu_{0}\Omega L}{2n_{4}\;(\Omega )}\iint {d\omega }_{1}\;{d\omega }_{2}{\tilde{\chi }}^{\left(3\right)}\;(\omega_{\mathrm{eg}}-\omega_{1},\;\omega_{1}-\omega_{2},\;-\omega_{\mathrm{eg}}+\Omega ){\tilde{E}}_{1}^{*}\;(z,\;\omega_{1}){\tilde{E}}_{2}\;(z,\omega_{2})\\ & \times {\tilde{E}}_{3}\;(z,\;\Omega -\omega_{2}+\omega_{1})\;\text{sin}\;c({\Delta k}_{z}\;(\Omega ,\;\omega_{1},\;\omega_{2})L/2)\text{exp}(i{\Delta k}_{z}\;(\Omega ,\;\omega_{1},\;\omega_{2})L/2) \end{array}$$

Here, Δ*k_z_* (Ω, *ω*_1_, *ω*_2_) = −*k*_1*z*_ (*ω*_1_) + *k*_2*z*_ (*ω*_2_) + *k*_3*z*_ (Ω − *ω*_2_ + *ω*_1_) − *k*_4*z*_ (Ω is the phase mismatch, L is length of medium. As a result, the obtained four-wave mixing signal intensity can be expressed:

(3.3) $$I^{\left(3\right)}\;{}_{4}(\Omega )=\frac{\varepsilon_{0}n_{4}(\Omega )Q(\Omega )}{c}{\left|{\tilde{E}}_{4}(L,\;\Omega )\right|}^{2}\;\propto \;R(\Omega )I_{4}^{\mathrm{ideal}}\;(\Omega )$$

where, c is the vacuum light speed. *n*_4_ (Ω) is the index of generated signal at frequency Ω. Q(Ω) is the response function of the detector. $I_{4}^{\mathrm{ideal}}(\Omega )$ is an ideal four-wave mixing signal intensity and can be expressed:

(3.4) $$I_{4}^{\mathrm{ideal}}(\Omega )={\left|\iint d\omega_{1}d\omega_{2}{\tilde{E}}_{1}^{*}(z,\;\omega_{1}){\tilde{E}}_{2}(z,\;\omega_{2}){\tilde{E}}_{3}(z,\;\Omega -\omega_{2}+\omega_{1})\right|}^{2}$$

and:

(3.5) $$R(\Omega )=\frac{Q(\Omega )\Omega^{2}}{n_{4}(\Omega )}\;\text{sin}\;c^{2}(\Delta k_{z}\;(\Omega ,\;\omega_{1},\;\omega_{2})L/2)$$

**Figure 38.**
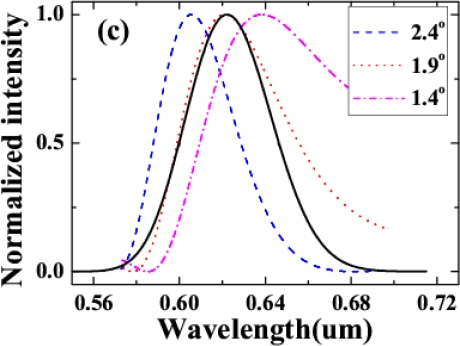
The black solid line is the ideal AS1 signal intensity. The dash-dot line, dotted line, and short-dashed line refer to curves related to phase mismatching sin *c*^2^ (Δ*k_z_* (Ω, *ω*_1_, *ω*_2_)*L*/2) at 1.4°, 1.9°, and 2.4°, respectively [33].

In the calculation, the input parameters are the same as above calculation. The ideal AS1 signal intensity was shown in Figure 38 with black solid line. The curves related to phase mismatching sin *c*^2^ (Δ*k_z_* (Ω, *ω*_1_, *ω*_2_)*L*/2) at 1.4°, 1.9°, and 2.4° were shown in Figure 38 with dash-dot line, dotted line, and short dash line, respectively. The same as above calculation, the phase mismatching was calculated through fixed one wavelength of beam1 and scan the wavelength of beam2 in several spectral bandwidth region in a 0.2-mm fused silica glass. It shows obviously the dependence of the spectrum of AS1 on the crossing angle of the two input beams. We can see that the output wavelength not only dependent on the phase mismatching but also the ideal curve (or the spectra of input pulses). In same way as described in references [54,66], $\frac{Q(\Omega )\Omega^{2}}{n_{4}(\Omega )}$ will also introduce blue-shift of the signal wavelength. As a result, the center wavelength of the generated sideband is not exact the wavelength that the phase mismatching is zero but a little shift to the center wavelength of the ideal spectrum.

## 5. Conclusions and Prospects

In conclusion, the CFWM process was theoretically analyzed and experimentally studied in bulk transparent media. Theoretical analysis and calculations based on the phase-matching condition taking into account the broadband spectra of the two incident pulses explained the process semi-quantitatively and cleared up the at first glance puzzling phenomena taking place in this process that explaining why the frequency difference between two adjacent sidebands decreased with increasing order number. As many as fifteen spectral up-shifted pulses and two spectral down-shifted pulses were obtained with spectral bandwidth broader than 1.8 octaves. The wavelengths of the sidebands are continuously tunable from near ultraviolet to near infrared by changing the crossing angle between the two input beams or replacing the nonlinear bulk medium. The obtained sidebands have good beam quality, with a M^2^ factor better than 1.1 and a nice Gaussian spatial profile. The pulse energy of the sideband can reach 1 μJ with power stability smaller than 1% RMS. As short as 15-fs compressed pulses, which were nearly transform-limited, were obtained when one of the two input beams was appropriately negatively chirped and the other was positively chirped. Moreover, broadband 2-D multicolored arrays with more than ten periodic columns and more than ten rows were generated when a sapphire plate was used as a nonlinear medium. By using XPM in conjunction with FOPA in another bulk medium, the obtained sideband was succeeded in smooth broadening of the weak pulse spectrum by a factor of about three and simultaneously amplifying the pulse energy by more than three times. Several weak beams with different wavelengths can be spectral broadened simultaneously at the same time in this way.

Several upshifted and downshifted multicolored femtosecond pulses can be simultaneously obtained and spatially separated from the input beams in the CFWM process. They are thus self-synchronized and convenient for multicolor pump-probe experiments [8], femtosecond CARS spectroscopy [9], and two-dimensional spectroscopy [10]. These wavelength tunable sidebands with good beam quality also suit for ultrafast nonlinear microscopy system [11–13].

In the future, CFWM sidebands could be obtained at much lower pulse energy incidence and higher repetition rate by using a medium with high third-order nonlinearity (for example nano-particle doped glass). This process can also be extended to the UV and Mid-IR spectral regions by using suitable input parameters. With the development of femtosecond fiber laser [67,68], this method can also be performed by using fiber laser system to make a compact system and even operate at a MHz repetition rate. Furthermore, these broadband sidebands can be used to obtain near single cycle pulse chain through Fourier synthesis of the sidebands. If the input beams are carrier-envelope phase (CEP) stabilized, all the generated sidebands would be CEP stabilized. As a result, CEP stabilized near single cycle pulse chain can be obtained in this way [28].

## Figures and Tables

**Table 1.** The output power of different sidebands for several different bulk media when the external crossing angle is about 1.8°. In the case, the input power of beam1 and beam2 are 7 mW and 25 mW, respectively [33].

μW	CaF_2_	Fused silica	BK7	Sapphire plate	BBO

AS1	480	700	715	750	780
AS2	210	315	295	210	135
AS3	125	90	60	40	10
